# Evolution of correlated complexity in the radically different courtship signals of birds-of-paradise

**DOI:** 10.1371/journal.pbio.2006962

**Published:** 2018-11-20

**Authors:** Russell A. Ligon, Christopher D. Diaz, Janelle L. Morano, Jolyon Troscianko, Martin Stevens, Annalyse Moskeland, Timothy G. Laman, Edwin Scholes

**Affiliations:** 1 Cornell Lab of Ornithology, Cornell University, Ithaca, New York, United States of America; 2 Department of Neurobiology and Behavior, Cornell University, Ithaca, New York, United States of America; 3 Centre for Ecology and Conservation, College of Life and Environmental Science, University of Exeter, Penryn, United Kingdom; 4 Museum of Comparative Zoology, Harvard University, Cambridge, Massachusetts, United States of America; University of Chicago, United States of America

## Abstract

Ornaments used in courtship often vary wildly among species, reflecting the evolutionary interplay between mate preference functions and the constraints imposed by natural selection. Consequently, understanding the evolutionary dynamics responsible for ornament diversification has been a longstanding challenge in evolutionary biology. However, comparing radically different ornaments across species, as well as different classes of ornaments within species, is a profound challenge to understanding diversification of sexual signals. Using novel methods and a unique natural history dataset, we explore evolutionary patterns of ornament evolution in a group—the birds-of-paradise—exhibiting dramatic phenotypic diversification widely assumed to be driven by sexual selection. Rather than the tradeoff between ornament types originally envisioned by Darwin and Wallace, we found positive correlations among cross-modal (visual/acoustic) signals indicating functional integration of ornamental traits into a composite unit—the “courtship phenotype.” Furthermore, given the broad theoretical and empirical support for the idea that systemic robustness—functional overlap and interdependency—promotes evolutionary innovation, we posit that birds-of-paradise have radiated extensively through ornamental phenotype space as a consequence of the robustness in the courtship phenotype that we document at a phylogenetic scale. We suggest that the degree of robustness in courtship phenotypes among taxa can provide new insights into the relative influence of sexual and natural selection on phenotypic radiations.

## Author summary

Animals frequently vary widely in ornamentation, even among closely related species. Understanding the patterns that underlie this variation is a significant challenge, requiring comparisons among drastically different traits—like comparing apples to oranges. Here, we use novel analytical approaches to quantify variation in ornamental diversity and richness across the wildly divergent birds-of-paradise, a textbook example of how sexual selection can profoundly shape organismal phenotypes. We find that color and acoustic complexity, along with behavior and acoustic complexity, are positively correlated across evolutionary timescales. Positive links among ornament classes suggests that selection is acting on correlated suites of traits—a composite courtship phenotype—and this integration may be partially responsible for the extreme variation in signal form that we see in birds-of-paradise.

## Introduction

Adaptive radiations are driven by ecological differences that promote processes of diversification and speciation [1]. In contrast, phenotypic radiations, which occur in the absence of clear ecological differentiation, are less well understood. One commonly investigated mechanism for phenotypic diversification among ecologically similar taxa is variation in social and sexual selection pressures promoting signal or ornament diversification. Ornamental radiations may come about as a consequence of variation in signaling environment [2,3], sensory capabilities [4,5], or pseudorandomly via mutation-order selection [6,7] or Fisher-Lande-Kirkpatrick processes [8–11]. Most studies investigating patterns of ornamental diversification have focused on individual trait classes and simplified axes of variation; however, sexual selection does not act on single traits in isolation. A more complete understanding of the processes driving ornamental diversification is possible only by investigating evolutionary relationships between the full suites of ornamental traits under selection.

Many animals rely on multiple ornamental traits to attract mates. Advantages of multiple ornaments may include increased information transfer (multiple messages), increased reliability (redundancy), increased flexibility (ensuring information transfer across contexts and environments), and increased memorability/discriminability [12–16]. Multiple ornaments may be more common when costs associated with the display or evaluation of those ornaments are low [17], as is likely the case in lekking species [12]. Though we now have broad empirical support for many of the proposed adaptive benefits of multiple signals at the level of individual species, how these specific hypotheses map onto our understanding of phylogenetic patterns of ornament evolution is less clear. Gaining insights into the macroevolutionary patterns of multiple ornament evolution is challenging, in part, owing to the difficulties of comparing highly divergent phenotypic traits across species. For instance, even focusing on evolutionary patterns of a single trait (e.g., plumage color in birds) across species can be difficult when traits possess different axes of variation (e.g., red versus blue). Though ingenious new methods have been devised to compare highly divergent ornaments of a single signal type (e.g., plumage color [18], electrical signals [19], or song [20]), comparing ornamental complexity across signal types presents yet an additional layer of complication. However, understanding the interrelationships of different classes of ornaments across phylogenetic scales can potentially provide valuable information about the evolutionary processes of communication, phenotypic radiation, and speciation that cannot be gathered from single-trait or single-species studies.

Following the evolution of multiple ornaments, selective pressures may favor different interrelationships among signal types. If ornamental investment is governed by evolutionary tradeoffs, investment in one class of ornaments will come only at the expense of investment in another. Evidence suggests that signal tradeoffs manifest as a negative correlation among ornament types across evolutionary time [21–27], reflecting strong, consistent constraints imposed by ecology, physiology, and natural selection [28,29]. Alternatively, instances in which ornamental traits show no evolutionary relationships [30–35] suggest long-term patterns of independent evolutionary trajectories. In such cases, signals are functionally independent and may even have evolved for use in different contexts (e.g., territorial defense versus mate attraction). When might we expect positive correlations among ornament classes across species? Theoretical [12,36] and empirical [37,38] work suggests that positive correlations among signals across species may reflect consistent selection acting similarly on separate axes of ornamental evolution. Strong, consistent intersexual selection could generate these positive correlations (sensu [37]), especially if the signals convey separate information [36], resulting in functional integration among ornament elements [39,40]. In such cases, positive correlations among signals across species would arise when selection favors an “integrated whole” of ornamental traits [41,42], which we call the “courtship phenotype.” The courtship phenotype is the composite expression of all ornamental classes evaluated during courtship and may represent the composite target of selection. Evolution may favor integrated, holistic mate evaluation strategies because of advantages that sensory overlap and redundancy offer (e.g., increased accuracy) [12–16].

**Fig 1:**
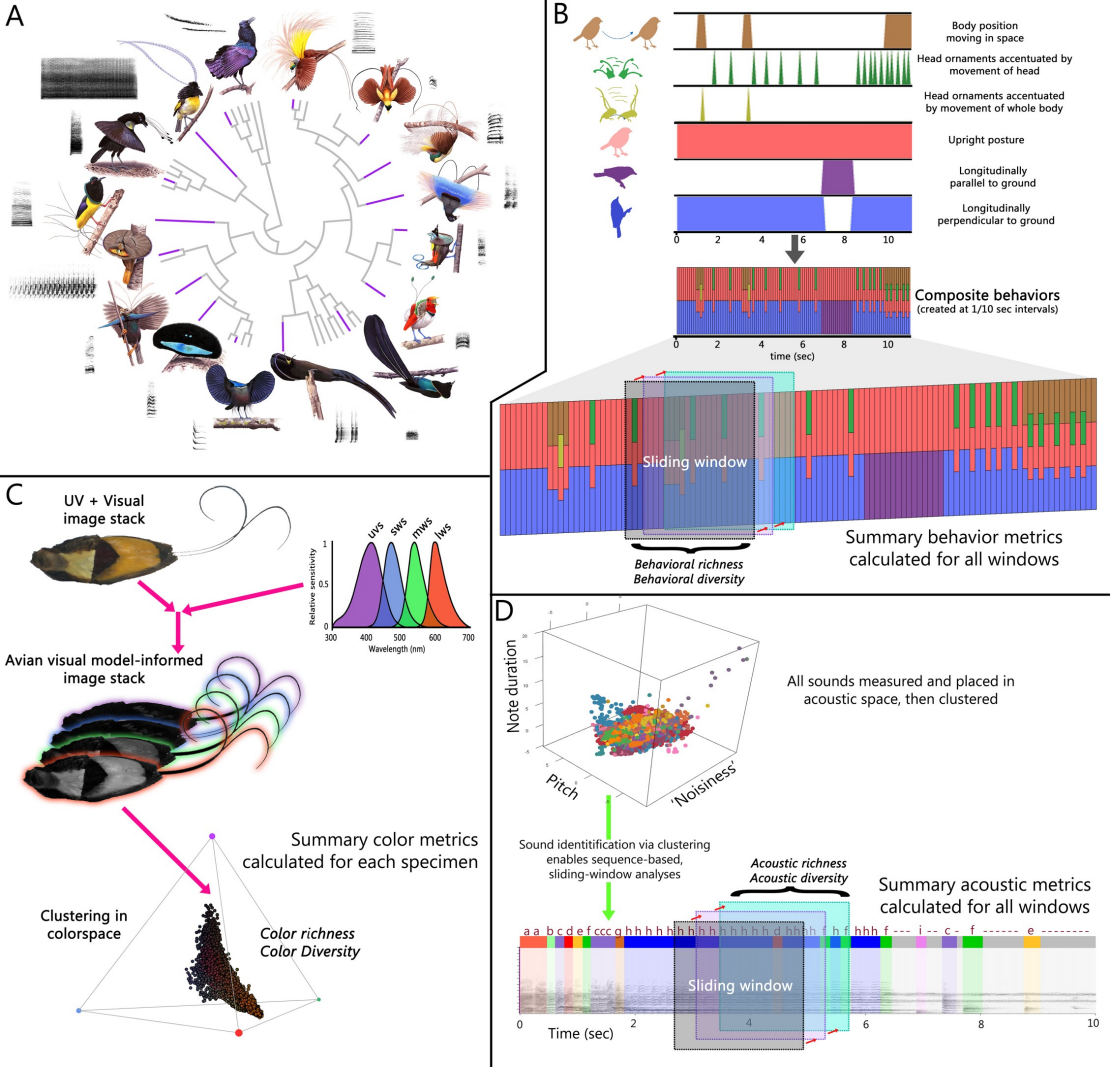
Birds-of-paradise exhibit extreme diversity in colors, sounds, and behaviors used during courtship displays, necessitating novel methods to quantitatively evaluate the evolution of their complexity. (A) Sixteen exemplar species (purple tips) are shown with their phylogenetic relationships to highlight variation in plumage color, acoustic signals, and courtship display behavior. (B) Behavioral subunits were scored from field-captured videos of displaying males (S3 and S4 Tables). Behavioral subunits were combined to create composite behaviors describing any behavior across species and facilitating sliding-window analysis of behaviors and behavioral sequences. (C) UV and visual spectrum images were taken of museum specimens (S7 Table) and used to generate avian visual model-informed image stacks. Color values were clustered with respect to modeled avian discriminability, enabling whole-specimen quantification of color richness and diversity. (D) All bird-of-paradise sounds were placed into a multidimensional acoustic space defined by principal components analysis. Sounds were then given identities based on locations within acoustic space, facilitating a sliding-window analysis of sounds and acoustic sequences (S8 and S9 Tables). lws, long-wavelength sensitive; mws, medium-wavelength sensitive; sws, short-wavelength sensitive; UV, ultraviolet; uvs, ultraviolet sensitive.

Here, we examine broad evolutionary patterns of ornamental signal investment and complexity across the wildly diverse [43,44], monophyletic [45] birds-of-paradise (Paradisaeidae) “…in which the process of sexual selection has gone to fantastic extremes” [46] (Fig 1A). We focus on the birds-of-paradise because this family exhibits extreme variation across species in multiple ornamental axes [43] (e.g., color [47–49] and behavior [50,51]) while possessing broadly similar life histories and mating systems [43,44]. Consequently, insights about the strength, direction, and diversification of ornamental phenotypes in this group may shed light on key processes of sexual selection and its power to generate phenotypic radiation when natural-selection–imposed constraints are minimized. In this study, we use a unique natural history dataset to quantitatively evaluate behavioral, acoustic, and colorimetric ornamentation across 40 species of birds-of-paradise, as well as relationships between signals and display environment.

## Results

### An approach to quantify courtship complexity among divergent ornaments

Comparisons across signal types are inherently challenging for evolutionary biologists given that such signals are necessarily measured in different ways. Additionally, comparisons within color, acoustic, and behavioral repertoires across taxa that vary widely (e.g., the birds-of-paradise) present an additional methodological challenge: how does one compare phenotypes that may share no obvious overlapping characters? We addressed this obstacle with a two-pronged approach to quantify ornamental complexity for behavior, color, and sounds in the birds-of-paradise. First, we broke down each ornament into a taxonomically unbounded character space that allowed classification of subunits across all species. Second, we used the specific attributes of a given ornament for each individual for each species to categorize the ornament components before quantifying two conceptually aligned measures of complexity for each signal type. Specifically, we evaluated richness (the number of unique elements) and diversity (using an index dependent on the number and relative contribution of each element type) using phylogenetic comparative approaches (see Methods for additional details).

For behavioral analyses, we first broke down the courtship behaviors of all species into distinct subunits shared across species (e.g., S1 Video). We then analyzed composite behavioral sequences across time using sliding-window analyses to compare maximally diverse behavioral repertoires for a set duration across species (Fig 1B). For colorimetric analyses, we relied on visual modeling of multispectral images to quantify the number and relative abundances of perceptually distinct color types across individuals and species. Though different colors may have different underlying production mechanisms, our analyses simply focused on the number and distribution of distinguishable colors (Fig 1C). Similar to our behavioral analysis pipeline, we used acoustic properties and agglomerative clustering to classify distinct sound types used by birds-of-paradise in courtship contexts before employing a similar sliding-window analysis to identify maximally diverse acoustic sequences, facilitating comparisons across species (Fig 1D).

**Fig 2:**
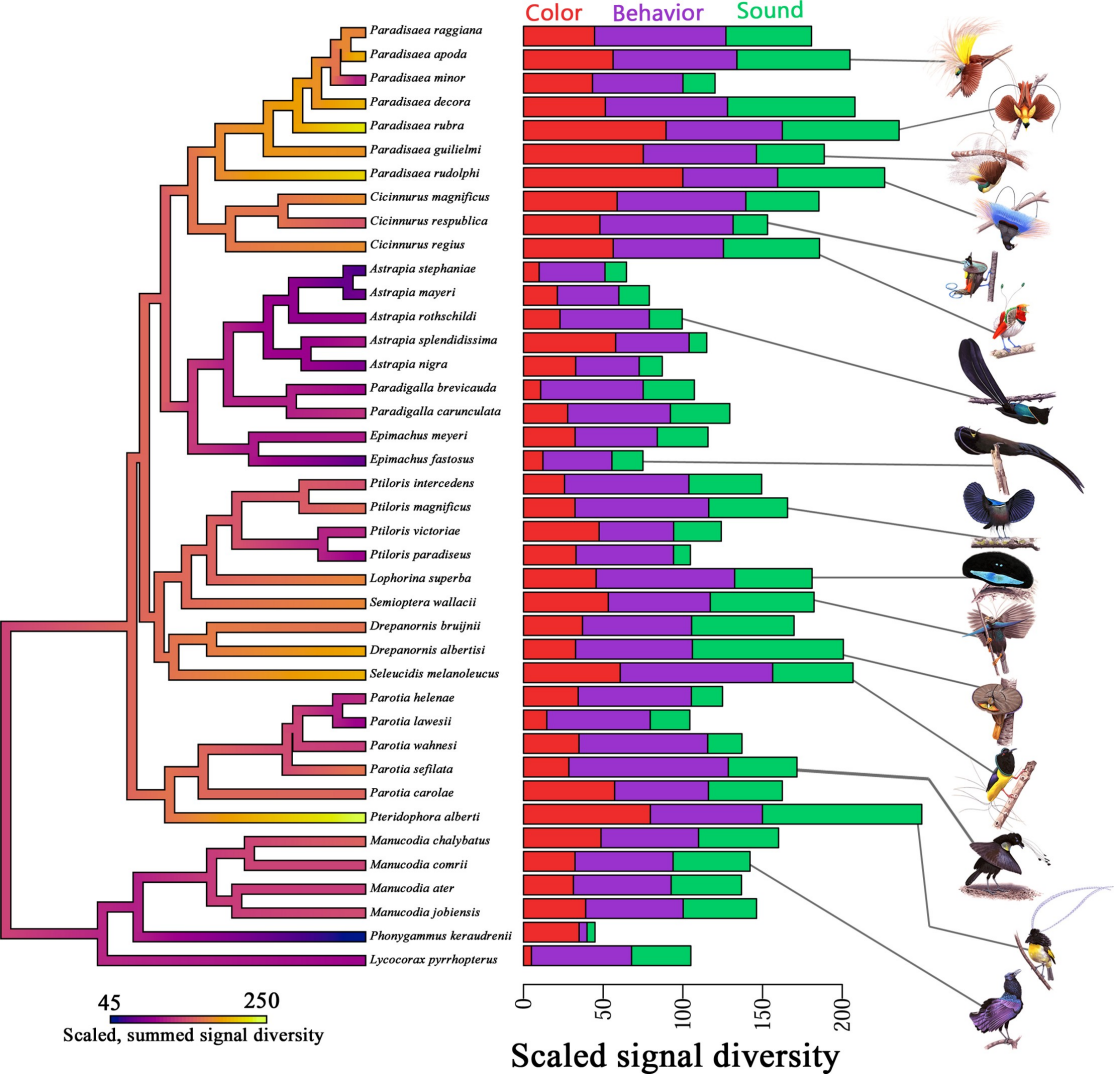
Signal diversity indices vary widely across birds-of-paradise. To facilitate interpretation of variation in signal diversity across the family (Paradisaeidae), we generated scaled diversity scores for each signal type (color, behavior, sound) to create (left) a composite metric of overall signal diversity (heat-mapped to the phylogeny) and (right) stacked bar plots illustrating variation in the relative diversity for each signal type for each species.

In total, we analyzed 961 video clips, 176 audio clips, and 393 museum specimens. From these analyses, we obtained quantitative diversity and richness metrics of ornamental complexity across the birds-of-paradise (Fig 2), which allowed us to rigorously evaluate patterns of correlated character evolution, as well as facilitating our investigation of the influence of breeding system and display environment on ornamental complexity.

### Integrative evolution of courtship complexity across modalities

**Fig 3:**
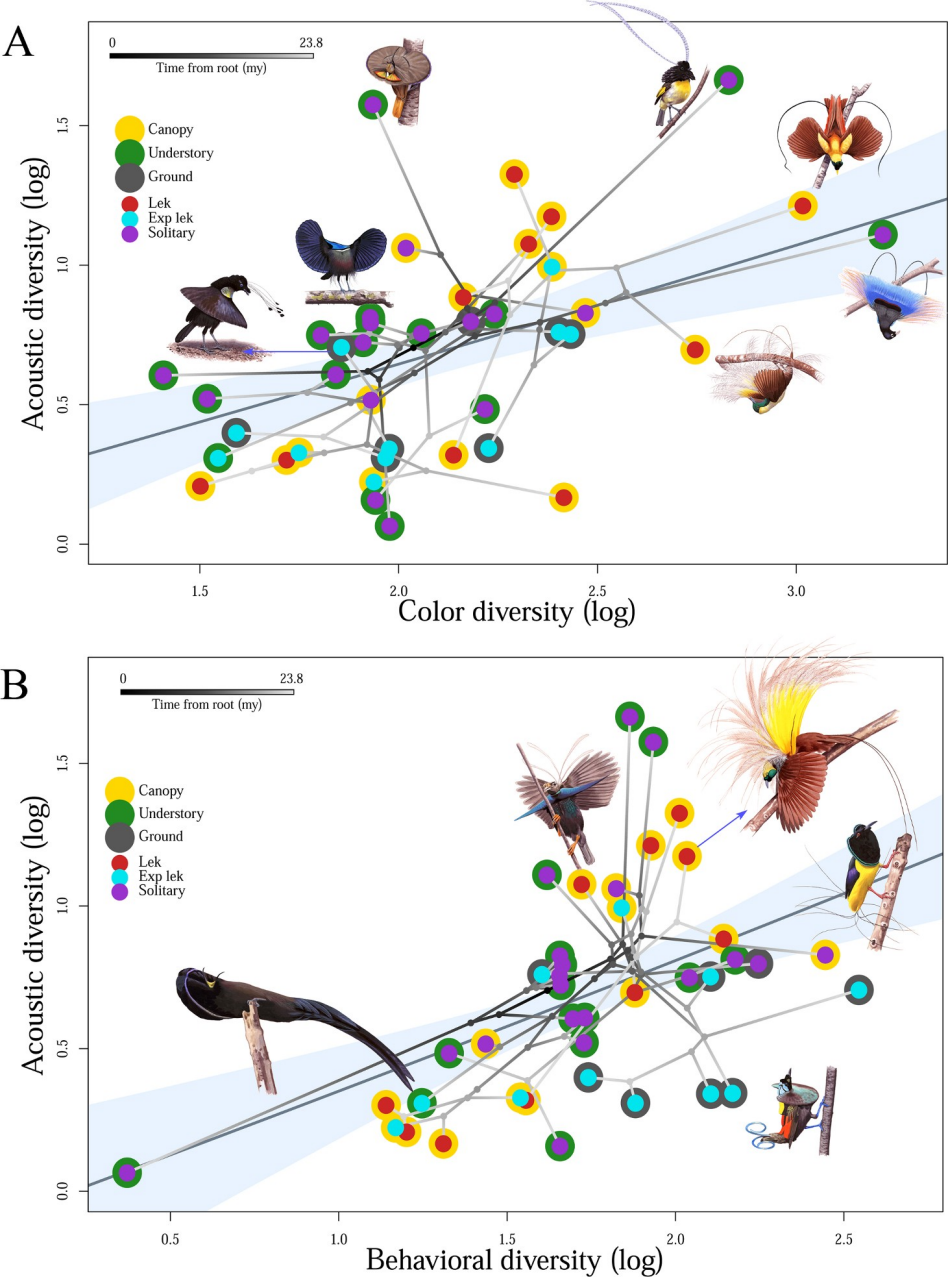
Positive phylogenetic correlations exist among several ornamental diversity indices at an evolutionary scale. (A) Color and acoustic diversity are positively correlated in the birds-of-paradise, where species with greater color diversity exhibit increased acoustic diversity when controlling for behavioral diversity, display height, and display proximity in an mPGLS regression (summary statistics in S1 Table). (B) Behavior and acoustic diversity are positively correlated in the birds-of-paradise, among whom species with greater behavioral diversity exhibit increased acoustic diversity when controlling for color diversity, display height, and display proximity in an mPGLS regression (summary statistics in S1 Table). Species’ points represent tip values for log transformed behavioral and color diversity. Underlying data for Fig 3 can be found in S1 Data. Exp lek, exploded lek; mPGLS, multiple phylogenetic generalized least squares; my, million years.

Using multiple phylogenetic generalized least squares (mPGLS) analyses, which allowed us to control for the nonindependence of species due to their shared evolutionary history, as well as the potentially confounding influences of display environment (both display height and proximity to courting conspecific males), we uncovered positive correlations between color and acoustic diversity (Fig 3A), as well as between behavioral and acoustic diversity (Fig 3B), consistent with the hypothesis that selection has acted similarly on these axes of ornamental complexity. Interestingly, however, there was no significant relationship between color diversity and behavioral diversity, indicating independent evolutionary trajectories for these visually encoded aspects of courtship ornamentation (S1 Table and S1 Fig). Analyses of ornamental richness revealed the same pattern to those uncovered for ornamental diversity (S2 Table). Specifically, behavioral richness and acoustic richness were correlated, as were color richness and acoustic richness (as was the case for both relationships involving ornamental diversity).

### Courtship complexity related to display height

**Fig 4:**
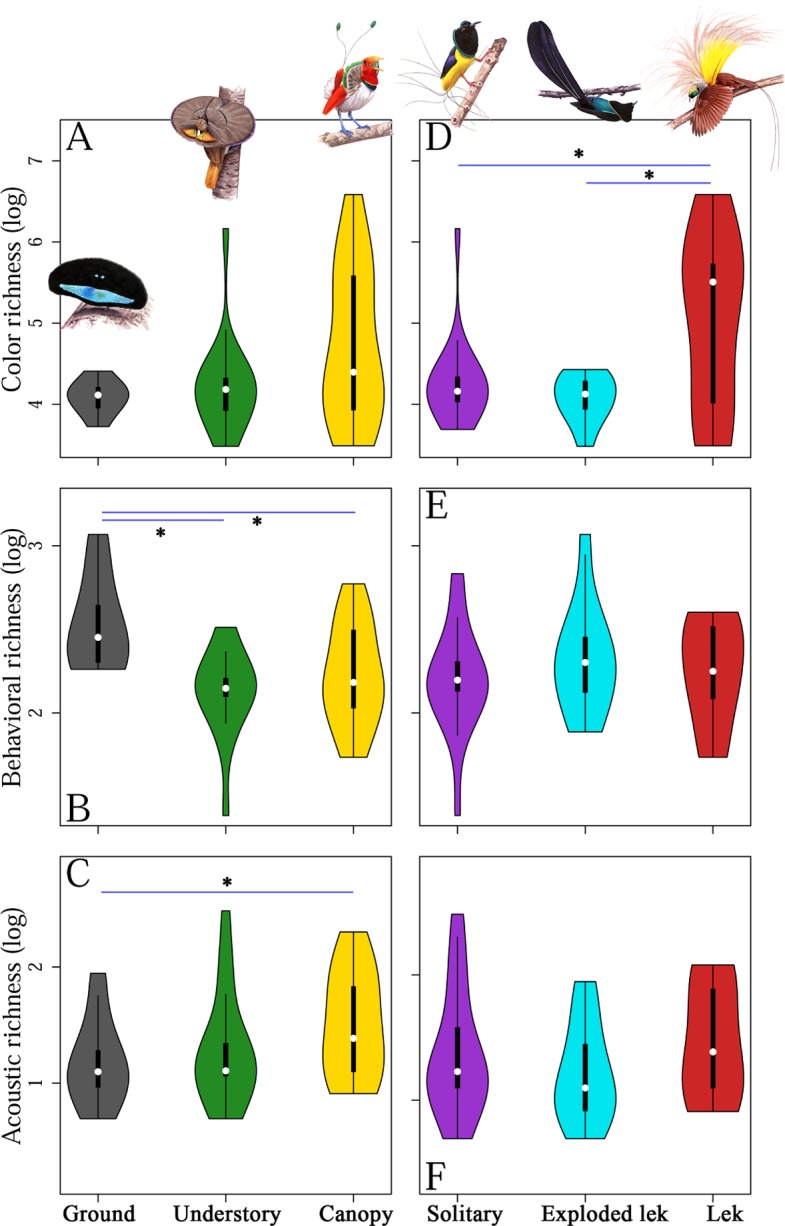
Social and environmental variation in display microhabitat influences multiple axes of ornamental complexity in birds-of-paradise. Display height did not influence color richness (A) but did influence behavioral (B) and acoustic (C) richness. Specifically, species that display on the forest floor have larger behavioral repertoires than species that display in the understory and canopy (B). Additionally, species that display in the canopy have larger acoustic repertoires compared to ground-displaying species (C). Social dynamics (D–F) of the display environment, measured as the proximity of other courting males, influenced color but not behavioral or acoustic richness in the birds-of-paradise. Species that display in classic leks have greater color richness (D) than species that display in exp leks or solitarily. However, neither behavioral (E) or acoustic (F) richness were significantly influenced by breeding/display system. Violin plots illustrate the distribution of log-transformed richness scores for each species. Underlying data for Fig 4 can be found in S1 Data. Exp lek, exploded lek.

Behavior richness and acoustic richness, but not color richness, were influenced by stratum of the forest in which species display (Fig 4A–4C). Specifically, we found that behavioral richness exhibited a negative relationship with display height among birds-of-paradise, such that species that display on the forest floor had the largest behavioral repertoires (S2 Table and Fig 4B). Species that display on the forest floor are typically operating with lower-light environments, and consequently, these species appear to rely more heavily on complex dance sequences to attract mates. Additionally, birds-of-paradise show increased acoustic (Fig 4C) richness as their display locations increase in height (S2 Table), a result that partially corresponds to the predictions of sensory drive [52,53] whereby the openness of the upper canopy favors increasingly complex acoustic displays.

Similar to the patterns we uncovered for signal richness, we also found that behavioral diversity and acoustic diversity were influenced by display height (S1 Table). Species displaying in the forest understory exhibiting a marginally significant (*p* = 0.051) trend for greater acoustic diversity relative to ground displaying species, and the behavioral diversity for ground-displaying species was higher than for both understory and canopy species (S1 Table). However, color diversity was not significantly influenced by display height.

### Courtship complexity related to spatial distribution of displaying males

Birds-of-paradise that display in classic leks have greater color richness (Fig 4D and S2 Table), corresponding to the increased strength of sexual selection on males to “stand out” visually when being evaluated simultaneously in lekking contexts. However, neither behavioral nor acoustic richness were significantly associated with the spatial distribution of displaying males. Furthermore, none of the diversity metrics (color, behavior, sound) were significantly associated with the breeding system structure (S1 Table).

## Discussion

Our study provides evidence that selection has favored correlated levels of ornamental diversity across multiple signals among the birds-of-paradise. This pattern of positive correlation among distinct ornament classes across evolutionary timescales and species suggests strong sexual selection on functionally integrated courtship phenotypes. The degree to which phenotypic traits are coexpressed and functionally dependent upon one another can be referred to as functional integration [54] or interdependence [40]. Courtship phenotypes with greater functional integration are therefore composed of ornaments that are typically expressed at similar levels and that are mutually interdependent in order to influence mate choice [39,41,42]. Correlations among the signals that comprise the courtship phenotype also suggest a previously undescribed robustness in bird-of-paradise courtship phenotypes that may have played a key role in the extreme ornamental radiation exhibited by this taxon (Fig 1).

Evolutionary biologists dating back to Mayr [55] and even Darwin [56] have recognized the potential evolutionary implications of functional redundancy (two or more structures performing the same function). Functional redundancy, including “true” redundancy (i.e., structurally identical components with identical functions) and degeneracy (i.e., structurally distinct components with similar functions) [57], facilitates evolutionary innovation (i.e., increases “evolvability”) by increasing robustness. Robust systems are those in which the overall structure and interconnectedness of parts provide protection from environmental or mutational instability [58] such that a given function is not lost if a single component fails. Robustness increases evolvability by enabling elements to react to selection independently and diverge while maintaining original functions [57,59]. All redundancy (both “true” redundancy and degeneracy) provides a measure of robustness, but robust systems are not necessarily redundant [60]. Given the broad theoretical [59,61,62] and empirical [63–65] support for the idea that robustness can promote evolvability across a wide array of biological domains, we posit that the correlations among signal types within birds-of-paradise courtship phenotypes are at least partially responsible for the dramatic diversification and radiation of courtship signals displayed by birds-of-paradise. If female birds-of-paradise make mate choice decisions based on sensory input from the multiple signals that comprise a composite courtship phenotype and information from those channels is correlated, then novel mutations changing the structure or form of a given ornament may occur without “necessary” information being lost [66]. Consequently, over evolutionary time, we suggest it is the inherent functional overlap (redundancy/degeneracy) and structural interdependency (robustness) of courtship phenotypes that leads to increased phenotypic diversification (evolvability) in birds-of-paradise.

Phenotypic radiations in the absence of clear ecological differentiation may arise stochastically [67,68] and be heavily influenced by the specific intricacies of female choice [7,10,69,70]. Birds-of-paradise clearly exhibit some ecological differentiation [43], but broadly speaking, they tend to be heavily frugivorous and predominantly polygynous [71]. They do not, however, all display to potential mates in the same contexts or microenvironments. Some species display high in the canopy, some down on the forest floor, and others in the understory in between. Likewise, some species display in large, cacophonous leks, some species in exploded leks (exp leks) in which males can hear but not see one another, and other species display solitarily. Our results suggest that these differences have shaped the specific courtship and signaling strategies of each species (Fig 4 and S1 and S2 Tables). Birds with richer acoustic repertoires display high in the canopy, where there is less environmental interference (e.g., from cluttered branches), increasing the likelihood that females will be able to detect and discern numerous, elaborate sounds [53]. Likewise, more behaviorally complex birds tend to display near the forest floor where there is less light (and ability to perceive subtle variation in color) but more area available for a courtship stage or “dance floor.” Birds that display in true leks have more colorful plumage, perhaps because females need to identify attractive individuals based on relatively unchanging traits, allowing them to compare among multiple displaying males simultaneously. Display site and display context thus influence the specific forms of ornamentation possessed by individual species [72], and taking them into account from an analytical perspective allows us to better understand patterns of signal coevolution and the potential importance of a functionally integrated courtship phenotype.

Signal efficacy and information content can exert strong influence on receiver preferences, and understanding both elements is integral when examining the evolution of complex, multicomponent courtship phenotypes [14,70,73,74]. The influence of receiver preference is difficult to overstate, particularly in birds-of-paradise, for which recent work indicates that selection acting on female preferences controls the rate, extent, and phenotypic space available for ornamental radiations [70]. Importantly, receiver preferences are influenced by the perceptual abilities [75,76] and psychology of signal receivers [77,78], as well as the environments through which signals are transmitted [52]—all of which can markedly influence signal efficacy. Additionally, the information content of multiple signals may increase the net amount of information transferred (e.g., multiple messages [16]) or increase accuracy and reliability if multiple signals communicate the same message (e.g., redundant signals [12,16]). The perceptual channels by which birds-of-paradise attract mates and those channels that are correlated at a phylogenetic scale provide tantalizing, though tentative, insights into the processes of efficient information transfer and receiver stimulation regulating mate choice in this group. Specifically, the fact that significant positive correlations exist between acoustic and color signals (auditory, visual), and between acoustic and behavioral signals (auditory, visual), but not between color and behavioral signals (visual, visual) aligns with psychometric literature on information and sensory input. When multiple sources of information are provided, information may be maximized if that information comes from separate channels (e.g., acoustic, visual) and lost when arriving through a single sensory channel [79] (but see [80]). What exactly this “information” might be in birds-of-paradise (quality [81], attractiveness [69], motivation [82], etc.) is not clear, but this result provides an interesting starting point for future investigations.

Phylogenetic comparative investigations of animal signals hold the potential to answer important questions about the evolutionary trajectories of communication over time [83,84]. However, the data used to tackle key questions of signal evolution necessarily place upper and lower bounds on the confidence and interpretations one can make from such comparative studies. It is our hope that the novel approaches we have developed to quantify color, sound, and behavior will be useful to other researchers interested in understanding signal variation at different scales. Though our primary aim was to generate methodological pipelines that facilitated comparisons among the highly divergent birds-of-paradise, the basic framework we describe here may also be useful for comparisons of more similar taxa—including studies of intraspecific variation in signaling effort (e.g., through sliding-window analyses focused on bouts of maximal complexity) or investment (e.g., by using receiver visual models to identify the number and perceptual similarity of color patches across individuals). Consequently, we feel that our approaches complement recent suggestions for incorporating a systems biology approach to the study of animal communication [57], wherein more comprehensive, higher-resolution data will only improve the validity and interpretability of analyses incorporating fitness surfaces and communication networks.

Evolutionary tradeoffs—increases in trait expression linked to reductions in another—are ubiquitous: “If there were no tradeoffs, then selection would drive all traits correlated with fitness to limits imposed by history and design” [85]. Tradeoff thinking can inform our interpretations of both the marked interspecific variation in overall signal complexity (Fig 2) and the finding that the ornaments of birds-of-paradise are positively correlated at phylogenetic scale (Fig 3). Firstly, interspecific variation in overall signal complexity suggests tradeoffs between investment in courtship and some other, unmeasured, variable that differs across species (e.g., microenvironment, paternal care, resource competition, etc.). Secondly, the absence of tradeoffs among signal types indicates an absence of differential costs on acoustic, behavioral, and chromatic signals. Further, the correlation among ornamental classes suggests that selection is acting on functionally integrated courtship phenotypes for birds-of-paradise, a finding that indicates female birds-of-paradise make mate choice decisions incorporating holistic, multicomponent information sets comprised of the various ornaments possessed by males of their species. Rather than being unique to birds-of-paradise, however, we suggest that this phenomenon is widespread among animals—though it is by varying degrees constrained, impeded, or obfuscated by conflicting and constraining processes and limitations imposed by ecology and natural selection. The degree to which selection has facilitated the evolution of integrated, robust courtship phenotypes may in fact serve as a proxy for the overall strength and consistency of female-driven sexual selection in any taxa, for which the integration and correlation among ornaments comprising the courtship phenotype may shed important light on the history and strength of sexual selection in that particular group.

## Methods

### Ethics statement

The study was focused on vertebrates (birds-of-paradise) but used museum specimens (physical and media), so no IACUC protocol was required.

### Behavioral complexity

We quantified the behavioral complexity of courtship display behaviors for the birds-of-paradise by scoring field-recorded video clips of 32 (80%) paradisaeid species, primarily from the Macaulay Library at the Cornell Lab of Ornithology (macaulaylibrary.org, S3 Table). In total, we watched 961 clips from 122 individuals totaling 47,707.2 s (approximately 795.12 min; mean clip duration = 49.64 s). Courtship display behavior is highly variable among bird-of-paradise species, necessitating broad behavioral categories to facilitate investigations of behavioral evolution. Specifically, one of us (CDD) blindly evaluated video clips of male birds-of-paradise displaying species-typical courtship behaviors [43] using a customized ethogram of behavioral units that enabled us to quantify all state and event behaviors exhibited by all species of Paradisaeidae (S4 Table).

#### Data collection

To record courtship display behaviors, we used a customized version of an open-source behavior logging program [86]. Additionally, we created a customized keyboard that allowed us to quickly and accurately record the start/stop times of all duration behaviors, as well as the instances of all event behaviors. The combinations of different behavioral categories throughout each clip allowed us to generate sequence data of distinct behavioral elements.

#### Measures of sexual display behavior complexity

Courtship displays can be broken down into distinct behavioral elements and the transitions between these elements. We investigated the number of unique behavioral elements (behavioral richness) in a given time period, as well as the Shannon entropy [87] of these behaviors (behavioral diversity). Shannon entropy provides a measure of “information” encoded in the behavioral displays, and we converted Shannon entropy scores to their numbers equivalents [88,89]. Shannon indices were chosen specifically because they are the only measures that “give meaningful results when community weights are unequal” [88]. As previously described [88], the numbers equivalent for Shannon entropy values has the readily interpretable property whereby a value of 2*x* would indicate a behavioral sequence with twice as many equally well-represented behaviors as a sequence with a value of *x*. In the context of behavioral displays, birds that use many unique behaviors and spend roughly equal amounts of time performing each display element (increased evenness as a proportion of time) will have higher diversity scores.

#### Sliding window analysis

The number of courtship recordings available was highly variable across species of birds-of-paradise (S3 Table). To reduce the influence of sampling intensity on our overall behavioral analyses, we used a sliding-window analysis to evaluate similar time windows for courtship display complexity across species. Specifically, we used a sliding 50 s window, chosen as the minimum duration resulting in relatively stable individual behavioral complexity scores (S2 Fig) across all clips for a given individual to identify the specific 50 s period of maximal display complexity for that individual, and incorporated the resultant complexity scores for this interval in our analysis. Individual scores were then averaged to obtain species-level estimates of signal complexity. Collectively, our approach minimizes the influence that variation in recording time and clip duration has on species-level behavioral comparisons. Our results and interpretations are robust to the choice of different window sizes between 10 and 60 s (S3 Fig, S4 Fig, S5 Table, and S6 Table).

### Color complexity

#### Image collection

We collected images from 393 bird-of-paradise museum specimens (S7 Table) housed at the American Museum of Natural History. Specifically, we took RAW format images of adult males from 40 bird-of-paradise species under standardized conditions using a Canon 7D camera (Tokyo, Japan) with full-spectrum quartz conversion and fitted with a Novoflex Noflexar 35 mm lens. Illumination was provided from two eyeColor arc lamps (Iwasaki, Tokyo, Japan) diffused through polytetrafluoroethylene sheets 0.5 mm thick. These arc lamps are designed to simulate International Commission on Illumination (CIE)-recommended daylight (D65) illumination (though they come standard with a UV-blocking coating, which we removed prior to use). Additionally, for every specimen position (see below), we used filters (Baader, Mammendorf, Germany) to take two photos, one capturing only UV light (300–400 nm) and one capturing wavelengths between 400 and 700 nm.

To simulate a variety of viewing angles and increase the likelihood of capturing relevant coloration from bird specimens, we took photographs of each specimen from three viewing angles: dorsal, ventral, and ventral angled. Specifically, each specimen was photographed from above while it was flat on its belly (dorsal view), flat on its back (ventral view), and angled 45° on its back (rotating the frontal plane along the vertical axis while keeping the head oriented in the same direction as the previous two photographs). The angled photograph was taken to increase the likelihood of capturing some of the variation made possible by iridescent plumage.

#### Image processing

UV and visible spectrum images were used to create standardized (i.e., channels were equalized and linearized [90]) multispectral image files for each specimen/position using the Image Calibration and Analysis Toolbox [91] in ImageJ [92].

- Avian color vision
  After estimating the color sensitivity of our camera/lens combination [90,91], we generated custom mapping functions to convert image colors to stimulation values corresponding to an avian visual space. Birds-of-paradise are inferred to have a violet-sensitive (VS) visual system [93], and the curl-crested manucode (*Manucodia comrii*) and magnificent riflebird (*Ptiloris magnificus*) have the same amino acid sequence in spectral tuning positions 84–94 [93] as the jackdaw (*Corvus monedula*) [94], which is inferred to have a peak sensitivity of its (VS-type) SWS1 cone at 408 nm. This sensitivity is similar to that of another species with a VS visual system, the pigeon *Columbia livia* (SWS1 peak sensitivity = 404 nm [95]). Consequently, we converted our full-spectrum photographs into the perceptual space of pigeons using physiological data [96], spectral sensitivity curve functions [97,98] (implemented in the R package *pavo* [99]), and multispectral imaging software [91] in ImageJ. Additionally, we evaluated color using a visual model from a UV-sensitive passerine species (the blue tit [100]) and found our results qualitatively unchanged. Prior to subsequent clustering (see below), we performed a median pixel blur to eliminate aberrant pixel values (owing to dust on the sensor, temporary dead pixels, etc.).
- Color clustering
  Following conversion to avian color vision and noise filtering, we used a novel custom-written agglomerative hierarchical clustering algorithm to reduce each multispectral image down to a perceptually relevant number of color clusters. This clustering algorithm was developed from a more basic algorithm used previously [101] that did not integrate luminance or thresholds when combining clusters. At the first step of the clustering process, each pixel is its own cluster. Each cluster is then compared to its neighboring clusters in the *XY* plane of the image within a given radius (1 pixel initially), and composite distances are calculated based on an equal weighting of chromatic [102] and achromatic [103] Just Noticeable Distances (JNDs) (using the log model). Specifically, chromatic and luminance JND values are divided by the chromatic JND threshold and luminance JND threshold, respectively, so that they are weighted equally based on the chosen threshold, and then the Euclidean distance of these two scaled values is calculated. Following distance calculations, each cluster is combined with its nearest (in composite JND distance) neighboring cluster if the composite difference is below the threshold for both luminance and chromatic JNDs. Nodes can have multiple clustering events at each pass, e.g., if cluster A is closest to B, but B is closest to C, and all distances are below the threshold, then all three will be clustered, meaning whole strings or neighboring regions can be clustered. In practice, each cluster tends to be combined with two or three clusters on each pass. On each pass, the updated mean cone catch values for each cluster are calculated, ready for the next pass. Additionally, the *XY* distance search radius increases with each pass, so as clusters get larger, they can also combine with neighboring clusters further away, which has the desirable effect of also keeping the processing for each pass relatively constant (i.e., there are fewer clusters on each pass, but each one must be compared to a larger number of neighbors). Clustering therefore takes place across *n* + 2 dimensions (*n* colors plus *x* and *y* space). The code used for this clustering is provided as supplementary material (S1 Code), and we have included a sample image illustrating the output of the clustering process (S5 Fig).
- Measures of color complexity
  Following color clustering, we quantified plumage color complexity using analogous indices to those we employed in our behavioral analysis. Namely, we quantified color richness (the number of distinct clusters) and color diversity (the numbers equivalent of Shannon index) for each view (dorsal, ventral, angled) and averaged these values to obtain individual, specimen-level metrics of color complexity. In terms of color, specimens with higher richness scores have more unique colors, and species with higher diversity scores have more, evenly distributed colors.
- Influence of specimen age on color complexity measures
  Aging can influence the coloration and appearance of some kinds of avian plumage [104,105], though such effects are often relatively small [106]. To evaluate the possibility that specimen age might influence our estimates of species’ level plumage elaboration, we conducted a linear mixed-effect model with two measures of color complexity (color richness, color diversity) as the dependent variable, collection year as the independent variable, and species as a random effect. Analyzing these models revealed no significant influence of collection year on either color richness (standardized *β* = 0.032, 95% CI −0.036 to 0.099, *t* = 0.930, *p* = 0.353) or diversity (standardized *β* = 0.033, 95% CI −0.012 to 0.080, *t* = 1.437, *p* = 0.151).

### Acoustic complexity

As with display behaviors, we quantified the acoustic complexity of courtship sounds produced by analyzing field-recorded audio/video clips of 32 (80%) bird-of-paradise species. In total, we analyzed sound from 176 clips from 59 individuals totaling 24,670.9 s (approximately 411 min; mean clip duration = 140.18 s; S8 Table). Though birds can generate sounds (both vocally and mechanically) in numerous contexts, we focused our analysis on recordings from known display sites or those matching written descriptions of courtship sound production [43].

#### Data collection

From each video clip used to quantify display behavior, we identified a focal individual and all of the sounds it produced. Spectrograms of the audio were viewed with a frequency resolution of 43.1 Hz and time resolution of 2.31 ms, and all sounds were marked in the sound analysis software RavenPro v. 1.5 [107]. Individual sounds were defined as temporally separated sound elements. Using the robust measurements in Raven, we measured the duration, maximum and minimum frequency, bandwidth, peak frequency, and peak frequency contour of each call. We measured the disorder, lack of organized or tonal structure, in a call with aggregate entropy and average entropy measures in Raven.

Following detailed analysis of the acoustic parameters for all notes, we used a two-step semiautomated classification analysis to assign note identity. In the first step, we conducted a principal component analysis (PCA) using 15 summary acoustic variables (S9 Table) followed by agglomerative hierarchical clustering to assign partial note identity (i.e., note classification based on location in PCA-based sound space). In the second step, each note was given a categorical identifier depending on the combination of four qualitative variables manually scored as yes/no (frequency modulation, nonharmonic structure, impulsive, stochastic). Full note identity was achieved by merging the clustered note identity with the combined qualitative categorization.

#### Measures of acoustic complexity

After assigning identities to all notes in our dataset, we measured acoustic richness (number of distinct note types) and acoustic diversity (Shannon index of notes) within a given time period (see Sliding window analysis below). As with behavior, we used the numbers equivalent of Shannon index values to facilitate more direct comparisons among samples and species.

#### Sliding window analysis

The duration and number of available courtship-specific acoustic recordings was highly variable across birds-of-paradise (S8 Table). To reduce the influence of this variation on species-level acoustic comparisons, we used a sliding-window analysis, similar to our behavioral analyses, to evaluate and compare similar time windows for acoustic display complexity across species. To identify the time period of maximal acoustic complexity for an individual in our analysis, we used a sliding 10 s window, chosen as the minimum duration resulting in relatively stable individual complexity scores (S6 Fig), across all clips for a given individual. Individual scores were then averaged to obtain species-level estimates of signal complexity. Relative complexity measures are robust to the choice of different window sizes between 5 and 50 s (S7 Fig and S8 Fig).

### Phylogenetic analyses

#### Tree

We regenerated a phylogenetic hypothesis from a recent molecular phylogeny for Paradisaeidae [45] using the function *phylo*.*tracer* in the R package *physketch* [108]. This ultrametric, timescaled tree was used for all downstream comparative analyses following one modification of tree topology. Specifically, we placed *Lophorina superba* as the outgroup to *Ptiloris* to accommodate a revised taxonomic hypothesis [109].

#### Phylogenetic generalized least squares

For each of the six components of courtship phenotype (color richness and diversity, behavior richness and diversity, acoustic richness and diversity), we conducted a single mPGLS regression evaluating the influence of the other elements of courtship phenotype and two signal-environment variables predicted to influence relative investment in separate axes of overall courtship phenotype. Specifically, we included a categorical metric of display height in all models, in which each species was scored as displaying on the forest floor, in the understory, or in the forest canopy. Additionally, we included the categorical metric of display proximity in all models, where each species was scored as displaying solitarily, in exp leks, or in true leks. In each model (see S1 Table and S2 Table), we included only “like” phenotype measures (e.g., including behavioral and acoustic richness but not behavioral or acoustic diversity when investigating the drivers of color richness). All courtship phenotype measures were log transformed prior to analyses, and analyses were performed in the R computing environment [110] using the *gls* function in the *nlme* package [111] assuming an Ornstein–Uhlenbeck (OU) covariance structure [112] using the *corMartins* function from the *ape* package [113].

#### Imputation

We used the *Rphylopars* package in R [114] to impute character values for taxa with missing data (e.g., species lacking behavioral/acoustic information, S1 Data). This methodology has previously been found to perform well in predicting ancestral and missing species’ values [115]. In our case, we evaluated the performance of several methods to estimate missing values assuming i) a Brownian motion model of trait evolution, ii) an OU model [112], iii) an “early-burst” model of trait evolution [116], iv) a Pagel’s lambda model of trait evolution [117], and v) a multivariate OU model [118]. We compared model performance by evaluating AIC scores and determined that the OU model performed best. Consequently, character trait values imputed using this model were used in all subsequent analysis.

Though data imputation can increase statistical power [119], the instances in which it might induce spurious findings are few, especially given the relatively small proportion of our total dataset (20%) for which we imputed values (cf. [120,121]). In fact, bias tends to be lower when missing data are imputed rather than omitted [115]. Regardless, to alleviate concerns that imputed values may drive subsequent findings, we also conducted our phylogenetic least squares (PGLS) analyses on the limited subset of species (*n* = 31) for which we have complete data. In all cases, the findings were qualitatively identical to those reported in the main text (S10 Table and S11 Table).

## Supporting information

S1 Video Bird-of-paradise behavioral scoring demonstration. **In this video, a male western parotia *Parotia sefilata* performs a species-typical courtship dance for females perching above.** This video demonstrates the concordance between a subsample of our scored behaviors and the actual performance of the bird in real-time. The behaviors represent BP1, BP2, SS1, O3, OPMH, OPMF, OPAH, and OPAC1. Users who cannot download the video can also view it here: https://youtu.be/MdqUO1RtbP0. BP1, body-position moving; BP2, changing direction while moving; OPAC1, ornamental flank plumage accentuation by moving the torso; OPAH, ornamental head plumage accentuation by moving the head; OPMF, ornamental flank plumage accentuation by moving those feathers; OPMH, ornamental head plumage accentuation by moving those feathers; O3, bowing; SS1, shape shifting.
(MP4)

S1 Code ImageJ plugin (java) for hierarchical clustering using chromatic and achromatic JNDs. JND, Just Noticeable Distance.
(JAVA)

S1 Data Species-specific courtship phenotype estimates. (XLS)

S2 Data Species-specific behavioral richness estimates from different sliding-window sizes (10 s–60 s). (XLS)

S3 Data Species-specific behavioral diversity estimates from different sliding-window sizes (10 s–60 s). (XLS)

S4 Data Species-specific acoustic richness estimates from different sliding-window sizes (5 s–50 s). (XLS)

S5 Data Species-specific acoustic diversity estimates from different sliding-window sizes (5 s–50 s). (XLS)

S1 Table mPGLS analyses of communication-relevant influences on three axes of courtship phenotype diversity. Categorical comparisons of display site are made with respect to ground-displaying birds, and breeding system comparisons are made with respect to solitarily displaying birds. mPGLS, multiple phylogenetic generalized least squares.
(DOCX)

S2 Table mPGLS analyses of communication-relevant influences on three axes of courtship phenotype richness. Categorical comparisons of display site are made with respect to ground-displaying birds, and breeding system comparisons are made with respect to solitarily displaying birds. mPGLS, multiple phylogenetic generalized least squares.
(DOCX)

S3 Table Species sampled for courtship behavior, including the number of individuals watched. (DOCX)

S4 Table Ethogram describing behavioral subunits scored while observing courtship display behavior of birds-of-paradise. (DOCX)

S5 Table mPGLS analyses of communication-relevant influences on three axes of courtship phenotype diversity conducted using behavioral complexity metrics from a 10 s and a 60 s time window. For comparison, the analyses presented in the main text focus on behavioral complexity estimated from a 50 s time window. mPGLS, multiple phylogenetic generalized least squares.
(DOCX)

S6 Table mPGLS analyses of communication-relevant influences on three axes of courtship phenotype richness conducted using behavioral complexity metrics from 10 s and 60 s time windows. The analyses presented in the main text focus on behavioral complexity estimated from a 50 s time window. mPGLS, multiple phylogenetic generalized least squares.
(DOCX)

S7 Table Summary of specimens located at the American Museum of Natural History used to quantify color complexity in the birds-of-paradise. (DOCX)

S8 Table Species sampled for acoustic courtship complexity, including the number of individuals analyzed. (DOCX)

S9 Table Partial summary (PC1–PC3) of PCA of 5739 notes produced by 32 bird-of-paradise species. PC loadings for PC1–PC3 were used to plot notes in three-dimensional PCA space prior to agglomerative hierarchical clustering based on Euclidean distances to categorize notes. PC, principal component; PCA, principal component analysis.
(DOCX)

S10 Table mPGLS analyses of communication-relevant influences on three axes of courtship phenotype diversity conducted only on species without imputed species level values. mPGLS, multiple phylogenetic generalized least squares.
(DOCX)

S11 Table mPGLS analyses of communication-relevant influences on three axes of courtship phenotype richness conducted only on species without imputed species level values. mPGLS, multiple phylogenetic generalized least squares.
(DOCX)

S1 Fig There is no evidence for correlated evolution between color and behavioral diversity among birds-of-paradise. mPGLS regression reveals no significant relationship between behavioral and color diversity when controlling for acoustic diversity, display height, and display proximity. This plot is a phylo-signal-space plot in which species ornamentation values are plotted with colored circles corresponding to display environment and mating system and are connected based on their phylogenetic relationships. Species’ locations represent tip values for log transformed behavioral and color diversity. Underlying data for S1 Fig can be found in S1 Data. mPGLS, multiple phylogenetic generalized least squares.
(DOCX)

S2 Fig Accumulation of unique behaviors plateaus by time windows of approximately 50 s for most species. Note, these are unique behaviors per individual (not per clip). This distinction is important because some individuals were recorded in several clips, but in the longer clips they might not be doing much behaviorally (leading to the initially surprising drop in unique behaviors at certain longer window sizes).
(DOCX)

S3 Fig Pairwise comparisons of behavioral richness (number of unique behaviors) estimates for windows between 10 and 60 s in duration. Within plots, each point represents a species in the family Paradisaeidae, with species-specific values obtained from rphylopars reconstructions incorporating intra- and interspecific variation. Best-fit lines in lower plots, as well as F and P values presented in corresponding upper-diagonal squares, come from PGLS analysis assuming OU error structure. Results are qualitatively identical assuming different correlation structures (e.g., Pagel, Brownian). Underlying data for S3 Fig can be found in S2 Data. OU, Ornstein–Uhlenbeck; PGLS, phylogenetic generalized least squares.
(DOCX)

S4 Fig Pairwise comparisons of behavioral diversity (Shannon indices of behaviors) estimates for windows between 10 and 60 s in duration. Within plots, each point represents a species in the family Paradisaeidae, with species-specific values obtained from rphylopars reconstructions incorporating intra- and interspecific variation. Best-fit lines in lower plots, as well as F and P values presented in corresponding upper-diagonal squares, come from PGLS analysis assuming OU error structure. Results are qualitatively identical assuming different correlation structures (e.g., Pagel, Brownian). Underlying data for S4 Fig can be found in S3 Data. OU, Ornstein–Uhlenbeck; PGLS, phylogenetic generalized least squares
(DOCX)

S5 Fig Dorsal view of raw (left side) and clustered (right side) images taken of a Wilson’s bird-of-paradise. Following clustering based on chromatic and achromatic thresholds (see Methods), every pixel in every image is assigned to a categorical color identity. The total number of colors in an image provides a measure of richness, and the numbers equivalent of the Shannon diversity of the colors—taking into account the relative area covered by each class of colors—provides a measure of color diversity. Individuals with higher richness scores have more colors, and individuals with more colors, more evenly distributed in terms of their relative areas, have higher diversity scores.
(DOCX)

S6 Fig Accumulation of unique notes plateaus at time windows of approximately 10 s for most species. (DOCX)

S7 Fig Pairwise comparisons of acoustic richness (number of unique note types) estimates for windows between 5 and 50 s in duration. Within plots, each point represents a species in the family Paradisaeidae, with species-specific values obtained from rphylopars reconstructions incorporating intra- and interspecific variation. Best-fit lines in lower plots, as well as F and P values presented in corresponding upper-diagonal squares, come from PGLS analysis assuming OU error structure. Results are qualitatively identical assuming different correlation structures (e.g., Pagel, Brownian). Underlying data for S7 Fig can be found in S4 Data. OU, Ornstein–Uhlenbeck; PGLS, phylogenetic generalized least squares.
(DOCX)

S8 Fig Pairwise comparisons of acoustic diversity (log transformed Shannon indices of acoustic complexity) estimates for windows between 5 and 50 s in duration. Within plots, each point represents a species in the family Paradisaeidae, with species-specific values obtained from rphylopars reconstructions incorporating intra- and interspecific variation. Best-fit lines in lower plots, as well as F and P values presented in corresponding upper-diagonal squares, come from PGLS analysis assuming OU error structure. Results are qualitatively identical assuming different correlation structures (e.g., Pagel, Brownian). Underlying data for S8 Fig can be found in S5 Data. OU, Ornstein–Uhlenbeck; PGLS, phylogenetic generalized least squares.
(DOCX)

## Abbreviations

CIE: international commission on illumination
exp lek: exploded lek
JND: Just Noticeable Difference
lws: long-wavelength sensitive
mPGLS: multiple phylogenetic generalized least squares
mws: medium-wavelength sensitive
my: million years
OU: Ornstein–Uhlenbeck
PCA: principal component analysis
PGLS: phylogenetic generalized least squares
sws: short-wavelength sensitive
UV: ultraviolet
uvs: ultraviolet sensitive
VS: violet sensitive
