# Supplementary material for: Evolution of correlated complexity in the radically different courtship signals of birds-of-paradise
Source: PLoS Biol. 2018 Nov 20;16(11):e2006962. doi: 10.1371/journal.pbio.2006962 (PMC6245505; doi:10.1371/journal.pbio.2006962)
Supplement: S1 Table — Categorical comparisons of display site are made with respect to ground-displaying birds, and breeding system comparisons are made with respect to solitarily displaying birds. mPGLS, multiple phylogenetic generalized least squares. (DOCX) [file pbio.2006962.s008.docx]

**S1 Table .** Multiple phylogenetic least-squares (mPGLS) analyses of communication-relevant influences on three axes of courtship phenotype diversity.

| Response variable | Predictor variable^1^ | Value | Std.Error | t-value | p-value^2^ |
| --- | --- | --- | --- | --- | --- |
| Color diversity (log) |  |  |  |  |  |
|  | (Intercept) | 2.12 | 0.44 | 4.78 | **0.000** |
|  | Behavioral diversity (log) | -0.17 | 0.20 | -0.83 | 0.410 |
|  | Acoustic diversity (log) | 0.65 | 0.19 | 3.37 | **0.002** |
|  | Understory display | -0.30 | 0.25 | -1.20 | 0.238 |
|  | Canopy display | -0.15 | 0.22 | -0.67 | 0.505 |
|  | Exploded lek | -0.07 | 0.20 | -0.34 | 0.734 |
|  | Classic lek | 0.10 | 0.21 | 0.49 | 0.629 |
|  |  |  |  |  |  |
| Behavioral diversity (log) |  |  |  |  |  |
|  | (Intercept) | 2.09 | 0.34 | 6.08 | **0.000** |
|  | Color diversity (log) | -0.12 | 0.15 | -0.83 | 0.410 |
|  | Acoustic diversity (log) | 0.65 | 0.15 | 4.22 | **0.000** |
|  | Understory display | -0.68 | 0.19 | -3.57 | **0.001** |
|  | Canopy display | -0.47 | 0.17 | -2.69 | **0.011** |
|  | Exploded lek | -0.16 | 0.17 | -0.93 | 0.360 |
|  | Classic lek | -0.12 | 0.18 | -0.70 | 0.492 |
|  |  |  |  |  |  |
| Acoustic diversity (log) |  |  |  |  |  |
|  | (Intercept) | -1.33 | 0.39 | -3.40 | **0.002** |
|  | Behavioral diversity (log) | 0.54 | 0.13 | 4.22 | **0.000** |
|  | Color diversity (log) | 0.40 | 0.12 | 3.37 | **0.002** |
|  | Understory display | 0.39 | 0.19 | 2.03 | *0.051* |
|  | Canopy display | 0.27 | 0.17 | 1.59 | 0.122 |
|  | Exploded lek | -0.06 | 0.16 | -0.36 | 0.724 |
|  | Classic lek | -0.02 | 0.16 | -0.10 | 0.922 |

1- Comparisons for categorical display height are made with respect to a ground-displaying species, and comparisons for categorical display proximity are made with respect to solitarily-displaying species.

2- Significant relationships are indicated by **bold** p-values, and those exhibiting non-significant trends indicated by *italicized* p-values.
