# Supplementary material for: Evolution of correlated complexity in the radically different courtship signals of birds-of-paradise
Source: PLoS Biol. 2018 Nov 20;16(11):e2006962. doi: 10.1371/journal.pbio.2006962 (PMC6245505; doi:10.1371/journal.pbio.2006962)
Supplement: S2 Table — Categorical comparisons of display site are made with respect to ground-displaying birds, and breeding system comparisons are made with respect to solitarily displaying birds. mPGLS, multiple phylogenetic generalized least squares. (DOCX) [file pbio.2006962.s009.docx]

**S2 Table**. Multiple phylogenetic least-squares (mPGLS) analyses of communication-relevant influences on three axes of courtship phenotype richness. Categorical comparisons of display site are made with respect to ground-displaying birds, and breeding system comparisons are made with respect to solitarily-displaying birds.

| Response variable | Predictor variable^1^ | Value | Std.Error | t-value | p-value^2^ |
| --- | --- | --- | --- | --- | --- |
| Color richness (log) |  |  |  |  |  |
|  | (Intercept) | 1.88 | 1.06 | 1.77 | 0.087 |
|  | Behavioral richness (log) | 0.57 | 0.41 | 1.41 | 0.168 |
|  | Acoustic richness (log) | 0.58 | 0.25 | 2.35 | **0.025** |
|  | Understory display | 0.38 | 0.44 | 0.87 | 0.389 |
|  | Canopy display | 0.15 | 0.41 | 0.37 | 0.714 |
|  | Exploded lek | 0.08 | 0.34 | 0.24 | 0.812 |
|  | Classic lek | 0.88 | 0.35 | 2.53 | **0.016** |
|  |  |  |  |  |  |
| Behavioral richness (log) |  |  |  |  |  |
|  | (Intercept) | 2.04 | 0.30 | 6.84 | **0.000** |
|  | Color richness (log) | 0.10 | 0.07 | 1.41 | 0.168 |
|  | Acoustic richness (log) | 0.22 | 0.10 | 2.12 | **0.042** |
|  | Understory display | -0.60 | 0.15 | -3.95 | **0.000** |
|  | Canopy display | -0.51 | 0.14 | -3.59 | **0.001** |
|  | Exploded lek | -0.19 | 0.14 | -1.42 | 0.164 |
|  | Classic lek | -0.10 | 0.16 | -0.63 | 0.533 |
|  |  |  |  |  |  |
|  |  |  |  |  |  |
| Acoustic richness (log) |  |  |  |  |  |
|  | (Intercept) | -1.16 | 0.70 | -1.66 | 0.107 |
|  | Behavioral richness (log) | 0.54 | 0.26 | 2.12 | **0.042** |
|  | Color richness (log) | 0.25 | 0.11 | 2.35 | **0.025** |
|  | Understory display | 0.24 | 0.29 | 0.82 | 0.416 |
|  | Canopy display | 0.51 | 0.25 | 2.05 | **0.049** |
|  | Exploded lek | -0.06 | 0.22 | -0.26 | 0.795 |
|  | Classic lek | -0.36 | 0.24 | -1.51 | 0.139 |

1- Comparisons for categorical display height are made with respect to a ground-displaying species, and comparisons for categorical display proximity are made with respect to solitarily-displaying species.

2- Significant relationships are indicated by **bold** p-values, and those exhibiting non-significant trends indicated by *italicized* p-values.
