# Supplementary material for: Evolution of correlated complexity in the radically different courtship signals of birds-of-paradise
Source: PLoS Biol. 2018 Nov 20;16(11):e2006962. doi: 10.1371/journal.pbio.2006962 (PMC6245505; doi:10.1371/journal.pbio.2006962)
Supplement: S3 Table — (DOCX) [file pbio.2006962.s010.docx]

**S3 Table**: Species sampled for courtship behavior, including the number of individuals watched.

| Species | N individuals | N clips | Mean duration of clips (s) | Total time watched (s) |
| --- | --- | --- | --- | --- |
| *Paradisaea raggiana* | 5 | 39 | 24.4 | 950 |
| *Paradisaea apoda* | 2 | 32 | 74.8 | 2392.8 |
| *Paradisaea minor* | 7 | 38 | 44.9 | 1706.3 |
| *Paradisaea rubra* | 7 | 76 | 21.9 | 1663.8 |
| *Paradisaea decora* | 4 | 40 | 56.5 | 2261.2 |
| *Paradisaea guilielmi* | 3 | 9 | 41.4 | 373.0 |
| *Paradisaea rudolphi* | 4 | 22 | 60.5 | 1330.8 |
| *Cicinnurus respublica* | 3 | 39 | 50.6 | 1973.1 |
| *Cicinnurus magnificus* | 4 | 30 | 73.3 | 2197.6 |
| *Cicinnurus regius* | 4 | 29 | 33.3 | 964.4 |
| *Astrapia mayeri* | 5 | 13 | 42.7 | 554.8 |
| *Astrapia rothschildi* | 3 | 20 | 69.0 | 1379.9 |
| *Astrapia splendidissima* | 1 | 4 | 74.1 | 296.4 |
| *Astrapia nigra* | 1 | 4 | 52.4 | 209.6 |
| *Epimachus meyeri* | 3 | 12 | 106.9 | 1282.5 |
| *Epimachus fastosus* | 5 | 25 | 223.3 | 5581.8 |
| *Ptiloris magnificus* | 3 | 20 | 152.1 | 3041.9 |
| *Lophorina superba* | 2 | 8 | 171.2 | 1369.8 |
| *Ptiloris victoriae* | 5 | 8 | 215.0 | 1719.7 |
| *Ptiloris paradiseus* | 5 | 21 | 35.4 | 742.8 |
| *Semioptera wallacii* | 6 | 60 | 15.3 | 917.5 |
| *Drepanornis bruijnii* | 2 | 3 | 69.7 | 209.1 |
| *Drepanornis albertisi* | 1 | 8 | 114.4 | 914.9 |
| *Seleucidis melanoleucus* | 1 | 19 | 48.2 | 915.3 |
| *Parotia wahnesi* | 6 | 174 | 24.7 | 4300.5 |
| *Parotia sefilata* | 2 | 54 | 44.4 | 2396.4 |
| *Parotia lawesii* | 7 | 51 | 34.3 | 1747.4 |
| *Parotia helenae* | 4 | 21 | 32.9 | 691.7 |
| *Parotia carolae* | 12 | 54 | 50.3 | 2714.5 |
| *Pteridophora alberti* | 3 | 24 | 31.8 | 763.3 |
| *Manucodia comrii* | 1 | 3 | 17.2 | 51.5 |
| *Phonygammus keraudrenii* | 1 | 1 | 92.9 | 92.9 |
