# Supplementary material for: Evolution of correlated complexity in the radically different courtship signals of birds-of-paradise
Source: PLoS Biol. 2018 Nov 20;16(11):e2006962. doi: 10.1371/journal.pbio.2006962 (PMC6245505; doi:10.1371/journal.pbio.2006962)
Supplement: S4 Table — (DOCX) [file pbio.2006962.s011.docx]

**S4 Table.** Ethogram describing behavioral subunits scored while observing courtship display behavior of birds-of-paradise.

| **Category** | **Description** | | | | |
| --- | --- | --- | --- | --- | --- |
| Body position | | |  | |  |
|  | Actively changing physical location | | | |  |
|  | While moving in space, changes direction of movement | | | |  |
|  | Actively moving towards female | | | |  |
| Shape-shifting | |  | | |  |
|  | Overall shape-shifted | | | |  |
|  | Partial shape-shifted | | | |  |
| Orientation | | |  | |  |
|  | Oriented towards female | | | |  |
|  | Changes orientation | | | |  |
|  | Inverted | | | |  |
|  | Upright | | | |  |
|  | Longitudinally parallel to ground | | | |  |
|  | Longitudinally perpendicular to ground | | | |  |
|  | Longitudinally parallel to display substrate | | | |  |
|  | Longitudinally perpendicular to display substrate | | | |  |
| Ornamental plumage movement | | | | |  |
|  | Ornamental head plumage accentuated by moving those feathers | | | |  |
|  | Ornamental back-of-neck plumage accentuated by moving those feathers | | | |  |
|  | Ornamental forehead/nostril plumage accentuated by moving those feathers | | | |  |
|  | Ornamental throat/breast plumage accentuated by moving those feathers | | | |  |
|  | Ornamental pectoral/flank plumage accentuated by moving those feathers | | | |  |
|  | Ornamental wing plumage accentuated by moving those feathers | | | |  |
|  | Ornamental tail plumage accentuated by moving those feathers | | | |  |
| Ornamental plumage accentuated by appendage movement | | | | |  |
|  | Ornamental head plumage accentuated by movement of head | | | |  |
|  | Ornamental throat/breast plumage accentuated by core(torso) movement (no foot movement) | | | |  |
|  | Back-of-neck ornament accentuation by movement of torso | | | |  |
|  | Ornamental pectoral/flank plumage accentuated by core(torso) movement (no foot movement) | | | |  |
|  | Ornamental pectoral/flank plumage accentuated by wing movement | | | |  |
|  | Ornamental wing plumage accentuated by moving torso | | | |  |
|  | Ornamental wing plumage accentuated by moving wings | | | |  |
|  | Tail ornaments accentuated by movements of the wing | | | |  |
|  | Tail ornaments accentuated by movements of the torso | | | |  |
| Ornamental plumage accentuated by whole body movement | | | | |  |
|  | Ornamental head plumage accentuated by whole-body movements | | | |  |
|  | Back-of-neck ornament accentuation by body | | | |  |
|  | Ornamental throat/breast plumage accentuated by whole-body movements | | | |  |
|  | Ornamental pectoral/flank plumage accentuated by whole-body movements | | | |  |
|  | Ornamental tail plumage accentuated by whole-body movements | | | |  |
|  | Ornamental wing plumage accentuated by whole-body movements | | | |  |
| Male initiated physical contact (not including contact immediately prior to copulation) | | | | |  |
|  | Head/bill touching | | | |  |
|  | Wing touching | | | |  |
|  | Flank plume touching | | | |  |
|  | Tail touching | | | |  |
| Prop use | | |  |  |  |
|  | Prop use | | | |  |
| Repetitive movements | | | | |  |
|  | Repetitive movement of wings that does *not* accentuate ornamental plumes | | | |  |
|  | Repetitive movement of tail that does *not* accentuate ornamental plumes | | | |  |
|  | Repetitive movement of legs that does *not* accentuate ornaments or cause locomotion | | | |  |
|  | Repetitive movement of head that does not accentuate ornaments or cause locomotion | | | |  |
|  | Repetitive torso movement | | | |  |
| Mouth opening | |  | | |  |
|  | Mouth is opened (wide) and closed rapidly | | | |  |
|  | Mouth is opened wide and held open | | | |  |

*Body part re-organization resulting in major changes to shape/size/complexity
