# Supplementary material for: Evolution of correlated complexity in the radically different courtship signals of birds-of-paradise
Source: PLoS Biol. 2018 Nov 20;16(11):e2006962. doi: 10.1371/journal.pbio.2006962 (PMC6245505; doi:10.1371/journal.pbio.2006962)
Supplement: S5 Table — For comparison, the analyses presented in the main text focus on behavioral complexity estimated from a 50 s time window. mPGLS, multiple phylogenetic generalized least squares. (DOCX) [file pbio.2006962.s012.docx]

**S5 Table.** Multiple phylogenetic least-squares (mPGLS) analyses of communication-relevant influences on three axes of courtship phenotype diversity conducted using behavioral complexity metrics from a 10s and 60s time-window. For comparison, the analyses presented in the main text focus on behavioral complexity estimated from a 50s time-window.

|  |  |  | (10s) | (60s) | (10s) | (60s) | (10s) | (60s) | (10s) | (60s) |
| --- | --- | --- | --- | --- | --- | --- | --- | --- | --- | --- |
| Response | Predictor^†^ |  | Value | Value | SE | SE | t-value | t-value | p-value | p-value |
| Color diversity (log) | |  |  |  |  |  |  |  |  |  |
|  | (Intercept) | | 1.95 | 1.94 | 0.43 | 0.45 | 4.56 | 4.34 | ***0.000** | ***0.000** |
|  | Behavioral diversity (log) | | -0.08 | -0.06 | 0.22 | 0.19 | -0.37 | -0.34 | 0.712 | 0.738 |
|  | Acoustic diversity (log) | | 0.60 | 0.59 | 0.20 | 0.19 | 2.99 | 3.10 | ***0.005** | ***0.004** |
|  | Understory display | | -0.24 | -0.25 | 0.24 | 0.26 | -0.99 | -0.94 | 0.330 | 0.354 |
|  | Canopy display | | -0.09 | -0.11 | 0.21 | 0.23 | -0.42 | -0.46 | 0.676 | 0.646 |
|  | Exploded lek | | -0.04 | -0.06 | 0.20 | 0.20 | -0.21 | -0.27 | 0.837 | 0.786 |
|  | Classic lek | | 0.12 | 0.11 | 0.21 | 0.21 | 0.58 | 0.55 | 0.569 | 0.586 |
|  |  |  |  |  |  |  |  |  |  |  |
| Behavioral diversity (log) | | |  |  |  |  |  |  |  |  |
|  | (Intercept) | | 1.71 | 2.05 | 0.32 | 0.36 | 5.31 | 5.62 | ***0.000** | ***0.000** |
|  | Color diversity (log) | | -0.05 | -0.05 | 0.14 | 0.16 | -0.37 | -0.34 | 0.712 | 0.738 |
|  | Acoustic diversity (log) | | 0.62 | 0.61 | 0.15 | 0.16 | 4.23 | 3.69 | ***0.000** | ***0.001** |
|  | Understory display | | -0.44 | -0.78 | 0.18 | 0.20 | -2.48 | -3.86 | ***0.018** | ***0.001** |
|  | Canopy display | | -0.23 | -0.55 | 0.16 | 0.19 | -1.43 | -2.96 | *****0.162 | ***0.006** |
|  | Exploded lek | | 0.04 | -0.18 | 0.16 | 0.18 | 0.26 | -0.98 | 0.793 | 0.336 |
|  | Classic lek | | 0.00 | -0.10 | 0.17 | 0.19 | 0.01 | -0.54 | 0.994 | 0.591 |
|  |  |  |  |  |  |  |  |  |  |  |
| Acoustic diversity (log) | |  |  |  |  |  |  |  |  |  |
|  | (Intercept) | | -1.18 | -1.21 | 0.37 | 0.40 | -3.19 | -3.00 | ***0.003** | ***0.005** |
|  | Behavioral diversity (log) | | 0.57 | 0.48 | 0.14 | 0.13 | 4.23 | 3.69 | ***0.000** | ***0.001** |
|  | Color diversity (log) | | 0.36 | 0.38 | 0.12 | 0.12 | 2.99 | 3.10 | ***0.005** | ***0.004** |
|  | Understory display | | 0.28 | 0.41 | 0.18 | 0.21 | 1.56 | 1.98 | 0.127 | *0.057* |
|  | Canopy display | | 0.15 | 0.28 | 0.16 | 0.18 | 0.91 | 1.56 | 0.370 | 0.129 |
|  | Exploded lek | | -0.16 | -0.07 | 0.15 | 0.16 | -1.07 | -0.41 | 0.292 | 0.684 |
|  | Classic lek | | -0.08 | -0.04 | 0.16 | 0.17 | -0.48 | -0.22 | 0.633 | 0.828 |

^†^ Comparisons for categorical display height are made with respect to a ground-displaying species, and comparisons for categorical display proximity are made with respect to solitarily-displaying species.

* Indicates significant relationships in the primary set of analyses incorporating behavioral complexity metrics drawn using 50s time windows.
