# Supplementary material for: Evolution of correlated complexity in the radically different courtship signals of birds-of-paradise
Source: PLoS Biol. 2018 Nov 20;16(11):e2006962. doi: 10.1371/journal.pbio.2006962 (PMC6245505; doi:10.1371/journal.pbio.2006962)
Supplement: S6 Table — The analyses presented in the main text focus on behavioral complexity estimated from a 50 s time window. mPGLS, multiple phylogenetic generalized least squares. (DOCX) [file pbio.2006962.s013.docx]

**S6 Table.** Multiple phylogenetic least-squares (mPGLS) analyses of communication-relevant influences on three axes of courtship phenotype richness conducted using behavioral complexity metrics from 10s and 60s time-windows. The analyses presented in the main text focus on behavioral complexity estimated from a 50s time-window.

|  |  |  | (10s) | (60s) | (10s) | (60s) | (10s) | (60s) | (10s) | (60s) |
| --- | --- | --- | --- | --- | --- | --- | --- | --- | --- | --- |
| Response | Predictor^†^ |  | Value | Value | SE | SE | t-value | t-value | p-value | p-value |
| Color richness (log) | |  |  |  |  |  |  |  |  |  |
|  | (Intercept) | | 2.29 | 2.03 | 0.82 | 1.05 | 2.80 | 1.93 | **0.009** | 0.062 |
|  | Behavioral richness (log) | | 0.54 | 0.50 | 0.39 | 0.39 | 1.40 | 1.28 | 0.170 | 0.211 |
|  | Acoustic richness (log) | | 0.57 | 0.59 | 0.25 | 0.25 | 2.28 | 2.36 | ***0.029** | ***0.024** |
|  | Understory display | | 0.20 | 0.31 | 0.38 | 0.43 | 0.52 | 0.73 | 0.610 | 0.471 |
|  | Canopy display | | 0.00 | 0.09 | 0.36 | 0.40 | 0.00 | 0.24 | 1.000 | 0.814 |
|  | Exploded lek | | -0.09 | 0.02 | 0.33 | 0.33 | -0.27 | 0.07 | 0.791 | 0.943 |
|  | Classic lek | | 0.77 | 0.83 | 0.35 | 0.35 | 2.19 | 2.38 | ***0.036** | ***0.023** |
|  |  |  |  |  |  |  |  |  |  |  |
| Behavioral richness (log) | | |  |  |  |  |  |  |  |  |
|  | (Intercept) | | 1.41 | 2.11 | 0.31 | 0.31 | 4.50 | 6.73 | ***0.000** | ***0.000** |
|  | Color richness (log) | | 0.10 | 0.09 | 0.07 | 0.07 | 1.40 | 1.28 | 0.170 | 0.211 |
|  | Acoustic richness (log) | | 0.25 | 0.25 | 0.11 | 0.11 | 2.30 | 2.28 | ***0.028** | ***0.029** |
|  | Understory display | | -0.31 | -0.55 | 0.16 | 0.16 | -1.94 | -3.48 | ******0.061* | ***0.001** |
|  | Canopy display | | -0.26 | -0.48 | 0.15 | 0.15 | -1.74 | -3.19 | ******0.091* | ***0.003** |
|  | Exploded lek | | 0.10 | -0.11 | 0.14 | 0.14 | 0.73 | -0.77 | 0.469 | 0.444 |
|  | Classic lek | | 0.08 | -0.01 | 0.16 | 0.16 | 0.50 | -0.05 | 0.622 | 0.962 |
|  |  |  |  |  |  |  |  |  |  |  |
| Acoustic richness (log) | |  |  |  |  |  |  |  |  |  |
|  | (Intercept) | | -0.83 | -1.20 | 0.57 | 0.68 | -1.45 | -1.76 | 0.156 | *0.087* |
|  | Behavioral richness (log) | | 0.55 | 0.55 | 0.24 | 0.24 | 2.30 | 2.28 | ***0.028** | ***0.029** |
|  | Color richness (log) | | 0.24 | 0.24 | 0.10 | 0.10 | 2.28 | 2.36 | ***0.029** | ***0.024** |
|  | Understory display | | 0.09 | 0.22 | 0.25 | 0.27 | 0.37 | 0.81 | 0.710 | 0.424 |
|  | Canopy display | | 0.37 | 0.49 | 0.22 | 0.24 | 1.64 | 2.03 | *****0.110 | ******0.051* |
|  | Exploded lek | | -0.21 | -0.10 | 0.21 | 0.21 | -1.03 | -0.46 | 0.311 | 0.646 |
|  | Classic lek | | -0.45 | -0.40 | 0.23 | 0.23 | -1.93 | -1.72 | 0.062 | 0.095 |

^†^ Comparisons for categorical display height are made with respect to a ground-displaying species, and comparisons for categorical display proximity are made with respect to solitarily-displaying species.

* Indicates significant relationships in the primary set of analyses incorporating behavioral complexity metrics drawn using 50s time windows.
