# Supplementary material for: Evolution of correlated complexity in the radically different courtship signals of birds-of-paradise
Source: PLoS Biol. 2018 Nov 20;16(11):e2006962. doi: 10.1371/journal.pbio.2006962 (PMC6245505; doi:10.1371/journal.pbio.2006962)
Supplement: S7 Table — (DOCX) [file pbio.2006962.s014.docx]

**S7 Table.** Summary of specimens located at the American Museum of Natural History used to quantify color complexity in the birds-of-paradise.

| Species | N | AMNH Specimen IDs |
| --- | --- | --- |
| *Paradisaea raggiana* | 10 | 330364,678807,678827,678820,678825,330363,350362,330365,330361,678804 |
| *Paradisaea apoda* | 10 | 268779,678750,678764,678737,678760,678770,678769,678762,678765,678768 |
| *Paradisaea minor* | 10 | 302427,678932,678936,678922,678940,678918,678925,678954,342217,678949 |
| *Paradisaea rubra* | 10 | 300993,678990,679001,300995,678991,300989,302703,678997,678999,295337 |
| *Paradisaea decora* | 12 | 784694,330384,330386,678973,678979,330383,330381,678975,678974,784693,678985,330380 |
| *Paradisaea guilielmi* | 10 | 303107,679025,679007,679008,679026,679006,679022,268187,268998,679024 |
| *Paradisaea rudolphi* | 11 | 679027,292159,248842,781619,679038,417400,679040,460899,809337,679042,679036 |
| *Cicinnurus respublica* | 10 | 408206,678497,300982,300984,678500,678490,292440,678493,460893,408205 |
| *Cicinnurus magnificus* | 11 | 678427,678422,294471,303143,303139,303141,303142,303145,294477,294476,840075 |
| *Cicinnurus regius* | 11 | 678610,835652,678606,678603,678520,678605,678609,835651,678608,678513,678611 |
| *Astrapia stephaniae* | 10 | 678054,678068,330355,678075,678058,678070,678061,330351,678069,678065 |
| *Astrapia mayeri* | 10 | 705535,705552,705539,705544,705554,705541,705533,705528,788307,705551 |
| *Astrapia rothschildi* | 10 | 678082,678079,678085,268983,823675,824219,678083,268984,268987,268985 |
| *Astrapia splendidissima* | 10 | 302952,302373,678026,678025,678022,342120,342105,678042,765907,765913 |
| *Astrapia nigra* | 10 | 678012,678017,678009,678013,678998,678011,678015,678016,678010,678018 |
| *Paradigalla carunculata* | 5 | 678333,789339,678337,678331,111201 |
| *Paradigalla brevicauda* | 10 | 678358,302982,302981,302983,678357,302980,678352,678353,678359,302979 |
| *Epimachus meyeri* | 10 | 677985,677987,677981,677984,677988,677975,677971,677976,677977,677967 |
| *Epimachus fastosus* | 10 | 677917,677912,677934,294544,294541,677909,677910,677919,677928,677918 |
| *Ptiloris magnificus* | 10 | 677545,677539,677521,294579,128771,294581,677528,294575,294572,677549 |
| *Ptiloris intercedens* | 10 | 268152,268158,268157,268153,268160,677571,677590,677591,677592,677597 |
| *Lophorina superba* | 10 | 678259,6787244,294590,294587,294589,294588,59359,678247,294386,266379 |
| *Ptiloris victoriae* | 10 | 677701,677702,677739,677703,677704,677705,677713,677711,677712,677710 |
| *Ptiloris paradiseus* | 10 | 677659,677653,677658,677652,677656,677661,677675,677655,677676,677683 |
| *Semioptera wallacii* | 10 | 467439,835517,678699,678697,678695,678706,678696,678698,467441,295338 |
| *Drepanornis bruijnii* | 13 | 677903,294563,111169,677889,677890,677904,677880,677875,677902,677899,677874,677884,677891 |
| *Drepanornis albertisi* | 11 | 330340,330339,677855,677856,677872,677852,677851,677870,677867,677853,677866 |
| *Seleucidis melanoleucus* | 10 | 677797,677770,677771,677792,677802,677781,677786,677791,677789,677796 |
| *Parotia wahnesi* | 4 | 268233,678237,678236,678238 |
| *Parotia sefilata* | 10 | 678099,678114,294606,678095,678108,678098,678106,678105,294608,678094 |
| *Parotia lawesii* | 10 | 272118,298259,784706,678209,330458,799759,678207,678214,809317,784705 |
| *Parotia helenae* | 3 | 678218,678224,678217 |
| *Parotia carolae* | 10 | 302972,678128,678134,678133,678144,678143,678159,678157,678158,678146 |
| *Pteridophora alberti* | 10 | 678676,302997,678678,678682,705644,705661,705646,705641,705642,705652 |
| *Manucodia comrii* | 10 | 330431,330430,784691,330436,677349,677348,216535,330453,330437,330439 |
| *Manucodia chalybatus* | 10 | 677313,677310,677312,294457,677311,677331,677319,268213,268214,268216 |
| *Manucodia jobiensis* | 10 | 302938,677296,303127,342018,303126,302357,302937,342033,830438,677248 |
| *Manucodia ater* | 10 | 301174,677249,300974,300975,677254,301176,791234,791235,830430,427583 |
| *Phonygammus keraudrenii* | 10 | 294446,677404,301181,301179,301180,427600,427601,427605,427598,427596 |
| *Lycocorax pyrrhopterus* | 12 | 8458,677479,677471,467412,467860,677472,467413,467859,467862,677484,467861,467858 |
