# Supplementary material for: Evolution of correlated complexity in the radically different courtship signals of birds-of-paradise
Source: PLoS Biol. 2018 Nov 20;16(11):e2006962. doi: 10.1371/journal.pbio.2006962 (PMC6245505; doi:10.1371/journal.pbio.2006962)
Supplement: S8 Table — (DOCX) [file pbio.2006962.s015.docx]

**S8 Table**. Species sampled for acoustic courtship complexity, including the number of individuals watched.

| Species | N individuals | N clips | Mean duration of clips (s) | Total time analyzed (s) |
| --- | --- | --- | --- | --- |
| *Paradisaea raggiana* | 1 | 3 | 94.8 | 284.3 |
| *Paradisaea apoda* | 1 | 8 | 68.0 | 543.8 |
| *Paradisaea minor* | 2 | 4 | 37.5 | 150.2 |
| *Paradisaea rubra* | 1 | 8 | 29.7 | 237.6 |
| *Paradisaea decora* | 2 | 6 | 28.6 | 171.6 |
| *Paradisaea guilielmi* | 1 | 3 | 4.1 | 12.3 |
| *Paradisaea rudolphi* | 1 | 2 | 58.1 | 116.3 |
| *Cicinnurus magnificus* | 3 | 18 | 146.4 | 2634.4 |
| *Cicinnurus respublica* | 2 | 4 | 36.8 | 147.3 |
| *Cicinnurus regius* | 2 | 3 | 72.3 | 216.9 |
| *Astrapia mayeri* | 1 | 1 | 26.2 | 26.2 |
| *Astrapia rothschildi* | 2 | 3 | 15.5 | 46.6 |
| *Astrapia splendidissima* | 1 | 4 | 53.4 | 213.7 |
| *Astrapia nigra* | 1 | 2 | 50.9 | 101.8 |
| *Epimachus meyeri* | 2 | 5 | 80.8 | 403.9 |
| *Epimachus fastosus* | 4 | 19 | 278.8 | 5297.1 |
| *Ptiloris magnificus* | 3 | 6 | 155.7 | 934.2 |
| *Ptiloris victoriae* | 1 | 2 | 633.5 | 1267.0 |
| *Ptiloris paradiseus* | 1 | 1 | 3.3 | 3.3 |
| *Lophorina superba* | 1 | 3 | 269.4 | 808.3 |
| *Semioptera wallacii* | 2 | 11 | 39.8 | 438.3 |
| *Drepanornis albertisi* | 1 | 4 | 304.7 | 1218.8 |
| *Seleucidis melanoleucus* | 1 | 3 | 60.2 | 180.5 |
| *Parotia wahnesi* | 6 | 9 | 370.6 | 3335.6 |
| *Parotia sefilata* | 2 | 20 | 140.7 | 2813.2 |
| *Parotia lawesii* | 2 | 5 | 79.9 | 399.6 |
| *Parotia helenae* | 1 | 1 | 52.1 | 52.1 |
| *Parotia carolae* | 7 | 11 | 200.6 | 2207.0 |
| *Pteridophora alberti* | 1 | 2 | 76.6 | 153.2 |
| *Manucodia comrii* | 1 | 3 | 9.6 | 28.7 |
| *Phonygammus keraudrenii* | 1 | 1 | 80.2 | 80.2 |
