# Supplementary material for: Evolution of correlated complexity in the radically different courtship signals of birds-of-paradise
Source: PLoS Biol. 2018 Nov 20;16(11):e2006962. doi: 10.1371/journal.pbio.2006962 (PMC6245505; doi:10.1371/journal.pbio.2006962)
Supplement: S9 Table — PC loadings for PC1–PC3 were used to plot notes in three-dimensional PCA space prior to agglomerative hierarchical clustering based on Euclidean distances to categorize notes. PC, principal component; PCA, principal component analysis. (DOCX) [file pbio.2006962.s016.docx]

**S9 Table.** Partial summary (PC1-PC3) of principal components analysis of 5739 notes produced by 32 BOP species. PC loadings for PC1-PC3 were used to plot notes in 3-dimensional PCA-space prior to agglomerative hierarchical clustering based on Euclidean distances to categorize notes.

|  | PC1 | PC2 | PC3 |
| --- | --- | --- | --- |
| Summary statistic |  |  |  |
| SD | 2.7348 | 1.6567 | 1.3759 |
| Proportion variance | 0.4986 | 0.183 | 0.1262 |
| Cumulate proportion of variance explained | 0.4986 | 0.6816 | 0.8078 |
|  |  |  |  |
| Variable |  | *loadings* |  |
| Duration (sec) | 0.07 | -0.21 | **0.64** |
| Robust Duration (sec) | 0.09 | -0.20 | **0.63** |
| ^1^Average entropy | **0.33** | -0.10 | -0.17 |
| ^2^Aggregate entropy | **0.33** | -0.11 | -0.14 |
| Min Entropy (bits) | **0.31** | -0.04 | -0.19 |
| Max Entropy (bits) | **0.32** | -0.12 | -0.05 |
| Bandwidth (Hz) | **0.33** | -0.14 | -0.10 |
| Peak Frequency (Hz) | 0.24 | **0.38** | 0.10 |
| Min Frequency (Hz) | 0.12 | **0.53** | 0.19 |
| Max Freq (Hz) | **0.34** | 0.12 | -0.01 |
| ^3^PFC Average Slope (Hz/ms) | 0.04 | -0.06 | **-0.18** |
| ^3^PFC Max Frequency(Hz) | **0.32** | 0.17 | 0.10 |
| ^3^PFC Max Slope (Hz/ms) | **0.27** | -0.24 | 0.03 |
| ^3^PFC Min Freq (Hz) | 0.12 | **0.54** | 0.12 |
| ^3^PFC Min Slope (Hz/ms) | **-0.27** | 0.20 | -0.08 |

^1^ Average entropy describes the amount of disorder for a typical spectrum within the selection by measuring the average of the entropy values for each time slice of the spectrogram.

^2^ Aggregate entropy measures the overall disorder in the sound by measuring the energy distribution in each frequency bin of the spectrogram.

^3^PFC = Peak frequency contour
