# Supplementary material for: Evolution of correlated complexity in the radically different courtship signals of birds-of-paradise
Source: PLoS Biol. 2018 Nov 20;16(11):e2006962. doi: 10.1371/journal.pbio.2006962 (PMC6245505; doi:10.1371/journal.pbio.2006962)
Supplement: S10 Table — mPGLS, multiple phylogenetic generalized least squares. (DOCX) [file pbio.2006962.s017.docx]

**S10 Table.** Multiple phylogenetic least-squares (mPGLS) analyses of communication-relevant influences on three axes of courtship phenotype diversity conducted only on species without imputed species level values.

| Response variable | Predictor variable^†^ | Value | Std.Error | t-value | p-value |
| --- | --- | --- | --- | --- | --- |
| Color diversity (log) |  |  |  |  |  |
|  | (Intercept) | 2.35 | 0.48 | 4.94 | ***0.000** |
|  | Behavioral diversity (log) | -0.17 | 0.22 | -0.79 | 0.436 |
|  | Acoustic diversity (log) | 0.50 | 0.20 | 2.46 | ***0.022** |
|  | Understory display | -0.28 | 0.28 | -1.00 | 0.329 |
|  | Canopy display | -0.12 | 0.22 | -0.55 | 0.591 |
|  | Exploded lek | -0.22 | 0.21 | -1.07 | 0.297 |
|  | Classic lek | 0.09 | 0.23 | 0.40 | 0.694 |
|  |  |  |  |  |  |
| Behavioral diversity (log) |  |  |  |  |  |
|  | (Intercept) | 2.20 | 0.46 | 4.73 | ***0.000** |
|  | Color diversity (log) | -0.16 | 0.20 | -0.79 | 0.436 |
|  | Acoustic diversity (log) | 0.63 | 0.17 | 3.64 | ***0.001** |
|  | Understory display | -0.78 | 0.22 | -3.61 | ***0.002** |
|  | Canopy display | -0.43 | 0.19 | -2.22 | ***0.036** |
|  | Exploded lek | -0.19 | 0.20 | -0.98 | 0.339 |
|  | Classic lek | -0.10 | 0.22 | -0.47 | 0.645 |
|  |  |  |  |  |  |
| Acoustic diversity (log) |  |  |  |  |  |
|  | (Intercept) | -1.53 | 0.54 | -2.84 | ***0.009** |
|  | Behavioral diversity (log) | 0.58 | 0.16 | 3.64 | ***0.001** |
|  | Color diversity (log) | 0.42 | 0.17 | 2.46 | ***0.022** |
|  | Understory display | 0.50 | 0.24 | 2.09 | **0.048** |
|  | Canopy display | 0.24 | 0.20 | 1.19 | 0.244 |
|  | Exploded lek | 0.02 | 0.20 | 0.12 | 0.904 |
|  | Classic lek | 0.07 | 0.21 | 0.34 | 0.740 |

^†^ Comparisons for categorical display height are made with respect to a ground-displaying species, and comparisons for categorical display proximity are made with respect to solitarily-displaying species.

*Indicates significant relationships in the ‘full’ analyses incorporating imputed character values.
