# Supplementary material for: Evolution of correlated complexity in the radically different courtship signals of birds-of-paradise
Source: PLoS Biol. 2018 Nov 20;16(11):e2006962. doi: 10.1371/journal.pbio.2006962 (PMC6245505; doi:10.1371/journal.pbio.2006962)
Supplement: S11 Table — mPGLS, multiple phylogenetic generalized least squares. (DOCX) [file pbio.2006962.s018.docx]

**S11 Table.** Multiple phylogenetic least-squares (mPGLS) analyses of communication-relevant influences on three axes of courtship phenotype richness conducted only on species without imputed species level values.

| Response variable | Predictor variable^†^ | Value | Std.Error | t-value | p-value |
| --- | --- | --- | --- | --- | --- |
| Color richness (log) |  |  |  |  |  |
|  | (Intercept) | 3.14 | 1.26 | 2.49 | **0.021** |
|  | Behavioral richness (log) | 0.19 | 0.48 | 0.40 | 0.692 |
|  | Acoustic richness (log) | 0.48 | 0.27 | 1.80 | **0.085* |
|  | Understory display | 0.15 | 0.51 | 0.30 | 0.768 |
|  | Canopy display | 0.03 | 0.43 | 0.08 | 0.939 |
|  | Exploded lek | -0.12 | 0.37 | -0.33 | 0.747 |
|  | Classic lek | 1.01 | 0.40 | 2.50 | ***0.020** |
|  |  |  |  |  |  |
| Behavioral richness (log) |  |  |  |  |  |
|  | (Intercept) | 2.28 | 0.39 | 5.86 | ***0.000** |
|  | Color richness (log) | 0.04 | 0.09 | 0.40 | 0.692 |
|  | Acoustic richness (log) | 0.24 | 0.11 | 2.17 | ***0.041** |
|  | Understory display | -0.67 | 0.17 | -3.90 | ***0.001** |
|  | Canopy display | -0.50 | 0.16 | -3.17 | ***0.004** |
|  | Exploded lek | -0.20 | 0.15 | -1.31 | 0.204 |
|  | Classic lek | 0.01 | 0.20 | 0.04 | 0.969 |
|  |  |  |  |  |  |
|  |  |  |  |  |  |
| Acoustic richness (log) |  |  |  |  |  |
|  | (Intercept) | -1.65 | 0.98 | -1.67 | 0.108 |
|  | Behavioral richness (log) | 0.70 | 0.32 | 2.17 | ***0.041** |
|  | Color richness (log) | 0.26 | 0.14 | 1.80 | **0.085* |
|  | Understory display | 0.42 | 0.36 | 1.15 | 0.264 |
|  | Canopy display | 0.55 | 0.30 | 1.85 | **0.077* |
|  | Exploded lek | 0.00 | 0.27 | -0.01 | 0.991 |
|  | Classic lek | -0.38 | 0.32 | -1.16 | 0.257 |

^†^ Comparisons for categorical display height are made with respect to a ground-displaying species, and comparisons for categorical display proximity are made with respect to solitarily-displaying species.

*Indicates significant relationships in the ‘full’ analyses incorporating imputed character values.
