# Supplementary material for: Evolution of correlated complexity in the radically different courtship signals of birds-of-paradise
Source: PLoS Biol. 2018 Nov 20;16(11):e2006962. doi: 10.1371/journal.pbio.2006962 (PMC6245505; doi:10.1371/journal.pbio.2006962)
Supplement: S1 Fig — mPGLS regression reveals no significant relationship between behavioral and color diversity when controlling for acoustic diversity, display height, and display proximity. This plot is a phylo-signal-space plot in which species ornamentation values are plotted with colored circles corresponding to display environment and mating system and are connected based on their phylogenetic relationships. Species’ locations represent tip values for log transformed behavioral and color diversity. Underlying data for S1 Fig can be found in S1 Data. mPGLS, multiple phylogenetic generalized least squares. (DOCX) [file pbio.2006962.s019.docx]

**
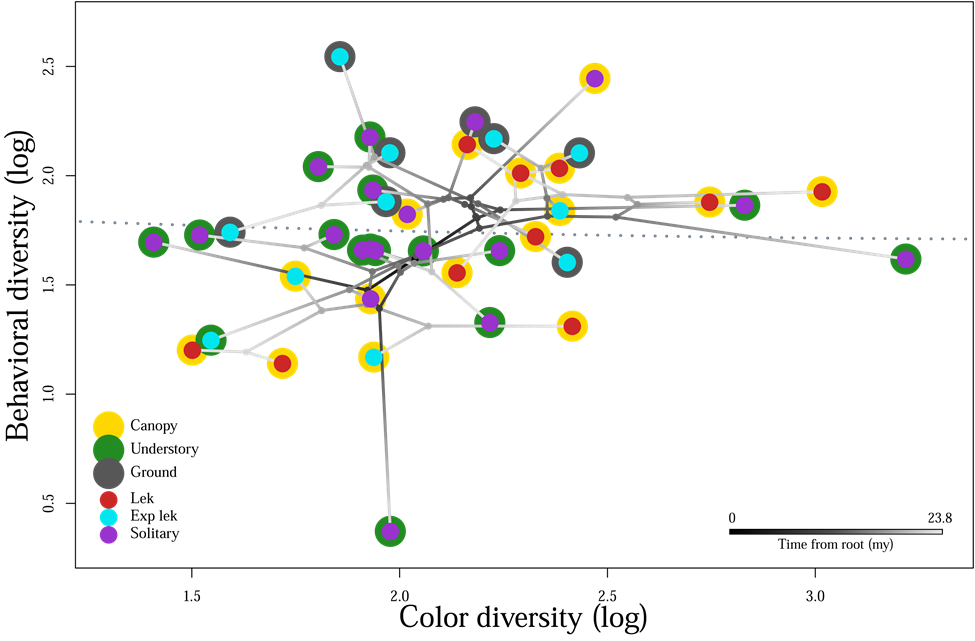
** **S1 Fig. There is no evidence for correlated evolution between color and behavioral diversity among birds-of-paradise**. Multiple phylogenetic least squares regression (mPGLS) reveals no significant relationship between behavioral and color diversity when controlling for acoustic diversity, display height, and display proximity. This plot is a phylo-signal-space plot where species ornamentation values plotted with colored circles corresponding display environment and mating system and are connected based on their phylogenetic relationships. Species’ locations represent tip values for log transformed behavioral and color diversity. Underlying data for S1 Fig can be found in S1 Data.
