# Supplementary material for: Evolution of correlated complexity in the radically different courtship signals of birds-of-paradise
Source: PLoS Biol. 2018 Nov 20;16(11):e2006962. doi: 10.1371/journal.pbio.2006962 (PMC6245505; doi:10.1371/journal.pbio.2006962)
Supplement: S2 Fig — Note, these are unique behaviors per individual (not per clip). This distinction is important because some individuals were recorded in several clips, but in the longer clips they might not be doing much behaviorally (leading to the initially surprising drop in unique behaviors at certain longer window sizes). (DOCX) [file pbio.2006962.s020.docx]

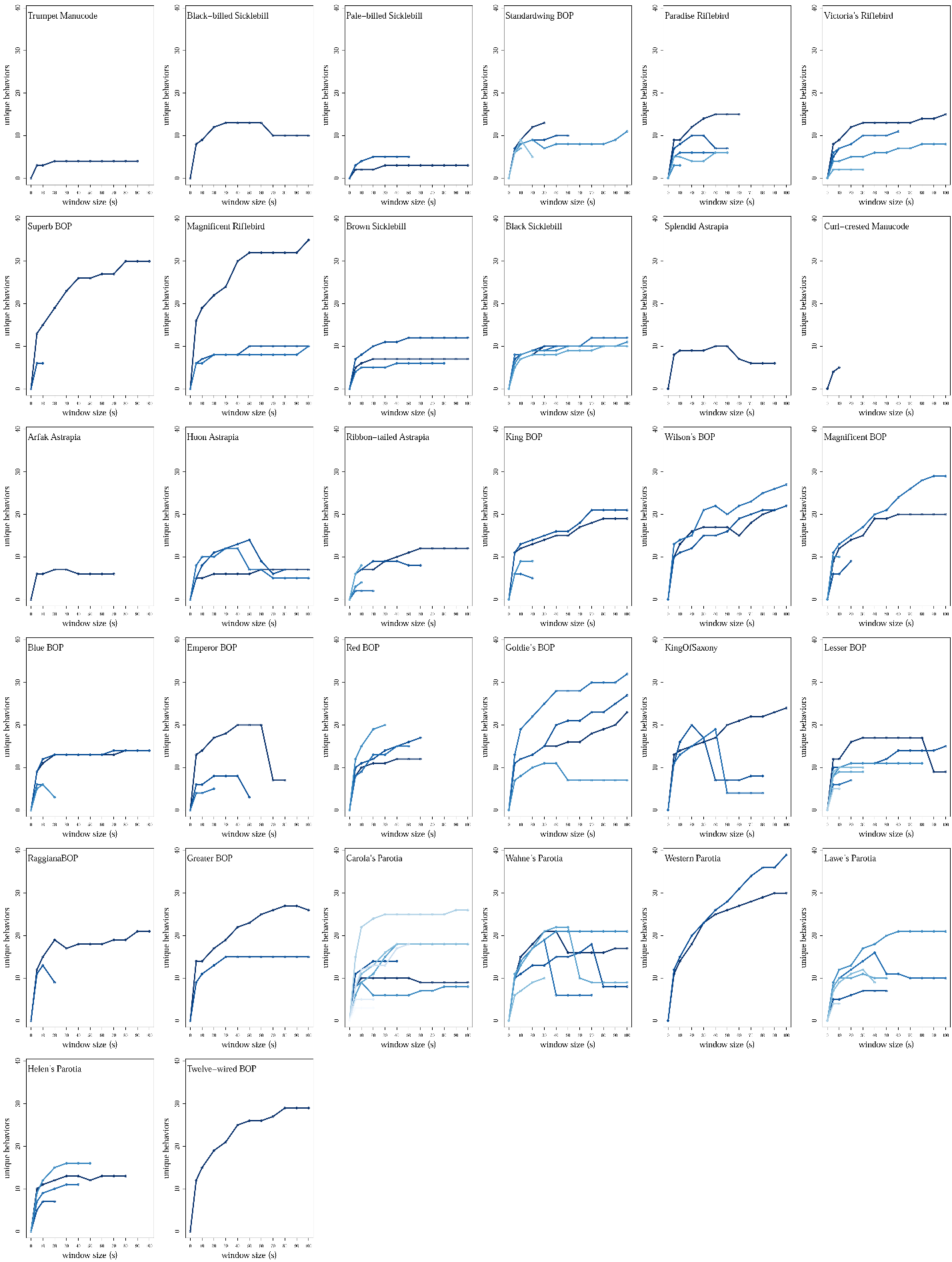


**S2 Fig.** Accumulation of unique behaviors plateaus by time windows of approximately 50 seconds for most species. Note, these are unique behaviors per individual (not per clip). This distinction is important because some individuals were recorded in several clips, but in the longer clips they might not be doing much behaviorally (leading to the initially surprising drop in unique behaviors at certain longer window sizes).
