# Supplementary material for: Evolution of correlated complexity in the radically different courtship signals of birds-of-paradise
Source: PLoS Biol. 2018 Nov 20;16(11):e2006962. doi: 10.1371/journal.pbio.2006962 (PMC6245505; doi:10.1371/journal.pbio.2006962)
Supplement: S5 Fig — Following clustering based on chromatic and achromatic thresholds (see Methods), every pixel in every image is assigned to a categorical color identity. The total number of colors in an image provides a measure of richness, and the numbers equivalent of the Shannon diversity of the colors—taking into account the relative area covered by each class of colors—provides a measure of color diversity. Individuals with higher richness scores have more colors, and individuals with more colors, more evenly distributed in terms of their relative areas, have higher diversity scores. (DOCX) [file pbio.2006962.s023.docx]

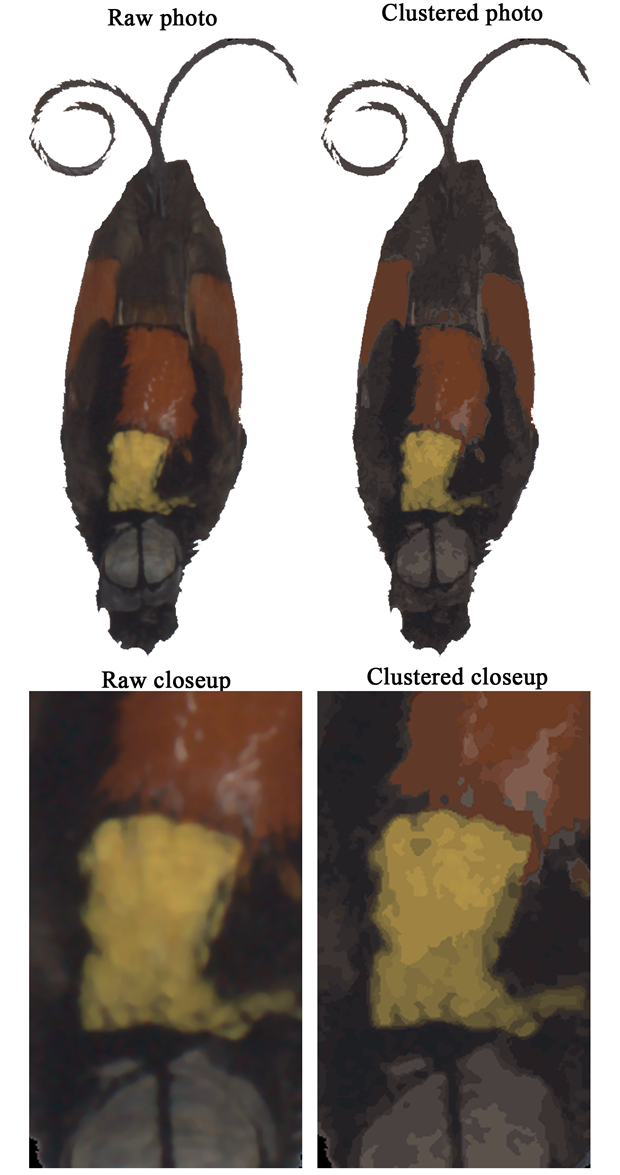


**S5 Fig. Dorsal view of raw (left side) and clustered (right side) images taken of a Wilson’s bird-of-paradise.** Following clustering based on chromatic and achromatic thresholds (see Methods), every pixel in every image is assigned to a categorical color identity. The total number of colors in an image provides a measure of richness, and the numbers equivalent of the Shannon diversity of the colors, taking into account the relative area covered by each class of colors, provides a measure of color diversity. Individuals with higher richness scores have more colors, and individuals with more colors, more evenly distributed in-terms of their relative areas, have higher diversity scores.
