# Supplementary material for: Evolution of correlated complexity in the radically different courtship signals of birds-of-paradise
Source: PLoS Biol. 2018 Nov 20;16(11):e2006962. doi: 10.1371/journal.pbio.2006962 (PMC6245505; doi:10.1371/journal.pbio.2006962)
Supplement: S6 Fig — (DOCX) [file pbio.2006962.s024.docx]

**
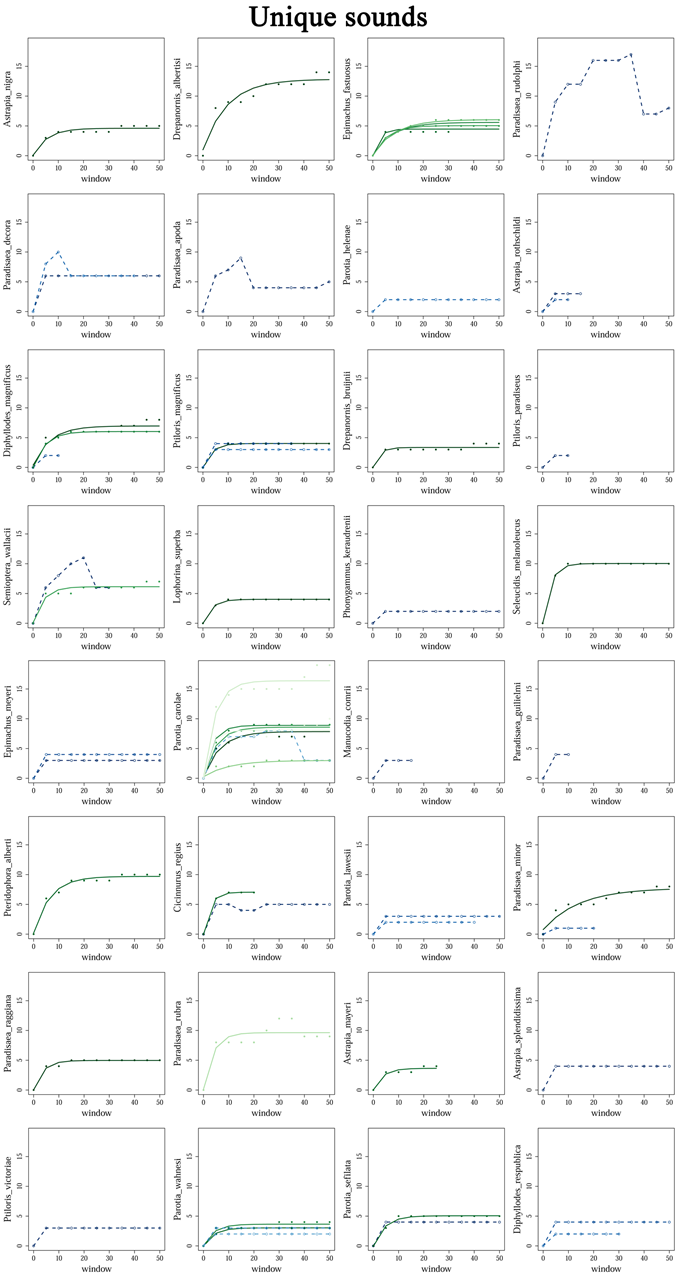
**

**S6 Fig.** Accumulation of unique sounds plateaus at time windows of approximately 10 seconds for most species.
