# Supplementary material for: Evolution of correlated complexity in the radically different courtship signals of birds-of-paradise
Source: PLoS Biol. 2018 Nov 20;16(11):e2006962. doi: 10.1371/journal.pbio.2006962 (PMC6245505; doi:10.1371/journal.pbio.2006962)
Supplement: S7 Fig — Within plots, each point represents a species in the family Paradisaeidae, with species-specific values obtained from rphylopars reconstructions incorporating intra- and interspecific variation. Best-fit lines in lower plots, as well as F and P values presented in corresponding upper-diagonal squares, come from PGLS analysis assuming OU error structure. Results are qualitatively identical assuming different correlation structures (e.g., Pagel, Brownian). Underlying data for S7 Fig can be found in S4 Data. OU, Ornstein–Uhlenbeck; PGLS, phylogenetic generalized least squares. (DOCX) [file pbio.2006962.s025.docx]

**
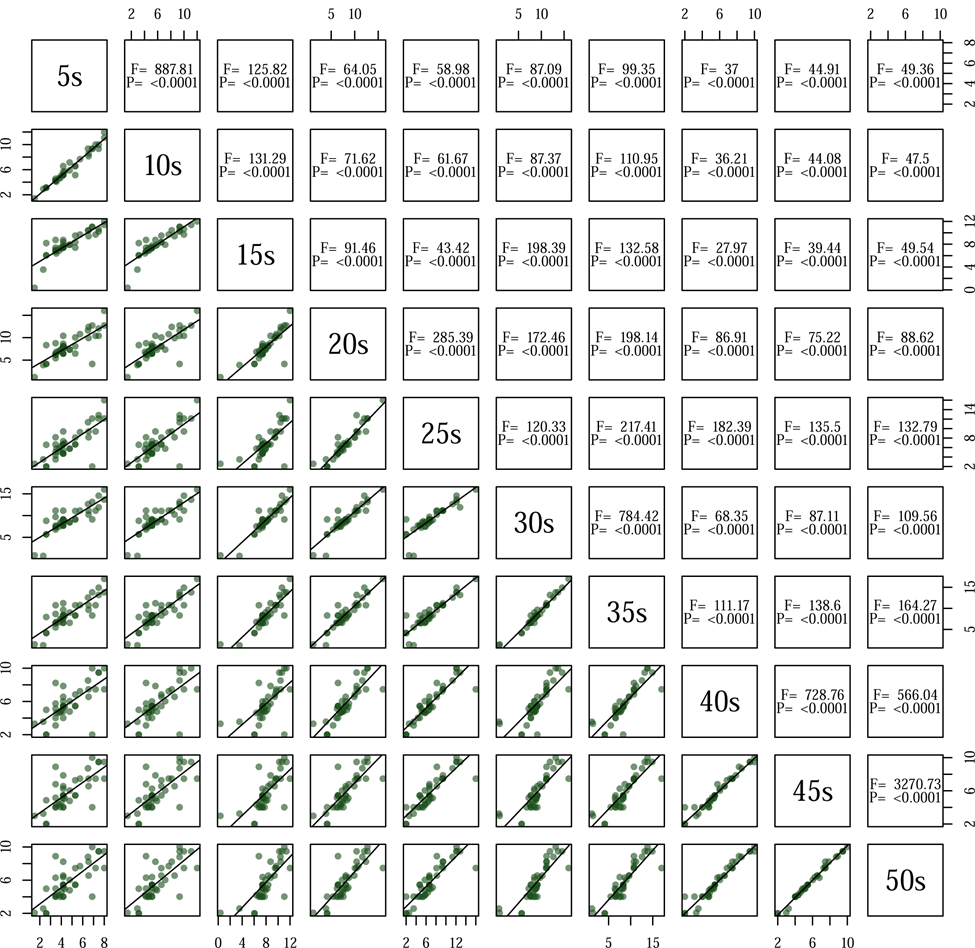
** **S7 Fig**. Pairwise comparisons of acoustic richness (number of unique note types) estimates for windows between 5 and 50 seconds in duration. Within plots, each point represents a species in the family Paradisaeidae, with species-specific values obtained from rphylopars reconstructions incorporating intra- and interspecific variation. Best-fit lines in lower plots, as well as F and P values presented in corresponding upper-diagonal squares, come from PGLS (phylogenetic generalized least squares) analysis assuming Ornstein–Uhlenbeck error structure. Results are qualitatively identical assuming different correlation structures (e.g. Pagel, Brownian). Underlying data for S7 Fig can be found in S4 Data.
